# PcG Proteins, DNA Methylation, and Gene Repression by Chromatin Looping

**DOI:** 10.1371/journal.pbio.0060306

**Published:** 2008-12-02

**Authors:** Vijay K Tiwari, Kelly M McGarvey, Julien D.F Licchesi, Joyce E Ohm, James G Herman, Dirk Schübeler, Stephen B Baylin

**Affiliations:** 1 Cancer Biology Division, The Sidney Kimmel Comprehensive Cancer Center, The Johns Hopkins University Medical Institutions, Baltimore, Maryland, United States of America; 2 Friedrich Miescher Institute for Biomedical Research, Basel, Switzerland; 3 Program in Cellular and Molecular Medicine, The Johns Hopkins University Medical Institutions, Baltimore, Maryland, United States of America; Adolf Butenandt Institute, Germany

## Abstract

Many DNA hypermethylated and epigenetically silenced genes in adult cancers are Polycomb group (PcG) marked in embryonic stem (ES) cells. We show that a large region upstream (∼30 kb) of and extending ∼60 kb around one such gene, *GATA-*4, is organized—in Tera-2 undifferentiated embryonic carcinoma (EC) cells—in a topologically complex multi-loop conformation that is formed by multiple internal long-range contact regions near areas enriched for EZH2, other PcG proteins, and the signature PcG histone mark, H3K27me3. Small interfering RNA (siRNA)–mediated depletion of EZH2 in undifferentiated Tera-2 cells leads to a significant reduction in the frequency of long-range associations at the *GATA*-4 locus, seemingly dependent on affecting the H3K27me3 enrichments around those chromatin regions, accompanied by a modest increase in *GATA*-4 transcription. The chromatin loops completely dissolve, accompanied by loss of PcG proteins and H3K27me3 marks, when Tera-2 cells receive differentiation signals which induce a ∼60-fold increase in *GATA-*4 expression. In colon cancer cells, however, the frequency of the long-range interactions are increased in a setting where *GATA-*4 has no basal transcription and the loops encompass multiple, abnormally DNA hypermethylated CpG islands, and the methyl-cytosine binding protein MBD2 is localized to these CpG islands, including ones near the gene promoter. Removing DNA methylation through genetic disruption of DNA methyltransferases (DKO cells) leads to loss of MBD2 occupancy and to a decrease in the frequency of long-range contacts, such that these now more resemble those in undifferentiated Tera-2 cells. Our findings reveal unexpected similarities in higher order chromatin conformation between stem/precursor cells and adult cancers. We also provide novel insight that PcG-occupied and H3K27me3-enriched regions can form chromatin loops and physically interact in *cis* around a single gene in mammalian cells. The loops associate with a poised, low transcription state in EC cells and, with the addition of DNA methylation, completely repressed transcription in adult cancer cells.

## Author Summary

Polycomb group (PcG) proteins and DNA methylation are fundamental epigenetic regulators of gene expression. The mechanisms underlying such regulation, the crosstalk between these mechanisms, and the role of higher order chromatin folding in mediating transcriptional control of involved genes remains unclear. Abnormal DNA methylation at gene promoters in cancer has been linked to PcG promoter occupancy and PcG-mediated maintenance of genes in a poised, low expression state in embryonic cells. We now strengthen these links and show that PcG occupancy around an entire gene, *GATA*-4, represses transcription by maintaining a series of long-range chromatin interactions. In embryonic cells, where DNA methylation is largely absent, *GATA*-4 is in a low, poised transcription state, and the loops can be virtually eliminated by retinoid-induced cellular differentiation, with attendant robust transcriptional up-regulation. When *GATA*-4 is DNA hypermethylated in colon cancer cells, the intensity of the long-range interactions is increased and associates with complete lack of transcription. Removal of DNA methylation in the cancer cells only slightly loosens the loops and restores expression to a low, poised state. Together, these findings suggest that both repressive pathways operate in part by the formation of chromatin higher order structures and provide important translational ramifications for targeting re-expression of epigenetically silenced genes for cancer therapy.

## Abstract 3

Chromatin regions enriched for Polycomb group proteins physically interact in a series of loops around a single gene in mammalian cells. This higher order structure maintains a poised, low transcription state in embryonic cancer cells and, with addition of DNA methylation, a completely repressed transcription in adult cancer cells.

## Introduction

Several mammalian Polycomb group (PcG) proteins play crucial roles during early embryonic development, and the PRC2 components EZH2 and EED were shown to be important for the self-renewal and pluripotency of embryonic stem (ES) cells [1–3]. Recently, a number of genome-wide analyses have uncovered several binding sites of PcG components in humans [4,5], mice [3], and Drosophila melanogaster [6–8]. These PcG target genes mainly encode transcriptional regulators that have a low basal transcription in pluripotent cells, which is often augmented during cell commitment to differentiation lineages [3,4,9–11]. The promoters of such genes can bear a combination of active, as well as the PcG inactive, histone modifications in ES cells that led to the proposal that these genes are held in a “transcription-ready” state and are available for induced expression upon receipt of specific developmental cues [11]. Thus, these so called “bivalent chromatin marks” are generally resolved during lineage commitment with diminution or loss of PcG marking upon transcriptional activation of involved genes. Global repression of key developmental regulatory genes by PcG proteins is, then, crucial for maintaining the pluripotency of ES cells when activation of the involved genes would otherwise lead to onset of differentiation.

The precise mechanisms underlying PcG-mediated repression of gene expression are not fully understood and are the focus of much investigation. Studies in D. melanogaster investigating the distribution pattern of PcG proteins and the associated histone marks along a locus have led to controversial results. Some show that PcG proteins are present at the PRE (Polycomb Response Element) and promoter regions, whereas H3K27 methylation is localized over the PRE [12–14], while others show PcG proteins are localized primarily to the PREs and me3K27 is spread over the entire gene [15]. Studies in mammalian cells clearly indicate that PcG proteins and associated H3K27me3 marks often occupy large chromatin domains sometimes spanning several kilobases of distance around the start sites of promoter CpG island containing genes or, occasionally, a group of genes [3–5,16–18]. These studies, however, do not clarify the functional importance of such broad distribution or how this might involve the interaction of PcG constituents over the area of occupancy.

The above issues are important to investigate in the context of the current exciting evolution in the understanding of transcriptional regulation. Long-range direct physical interactions between genes and regulatory elements including enhancers, silencers and chromatin insulators, in cooperation with distinct regulatory proteins, establish and maintain domains of gene expression or repression or “chromatin hubs” in a development- and differentiation-dependent fashion, which appear crucial for efficient transcriptional regulation [19–29]. It is unclear how PcG proteins contribute to such domains in mammalians cells. Long-range chromatin interactions by chromatin looping are thought to be one of the potential mechanisms to explain PcG action over broad distances in *cis* [15,30–33], but so far, no direct evidence has been put forth for mammalian cells. Very recently, Lanzuolo et al. provided the first direct evidence in *Drosophila* that, in the repressed state, all major PcG-bound elements at the BX-C multi-gene locus—including PREs and core promoters—physically associate by chromatin long-range interactions [34]. Evidence from studies in D. melanogaster as well as recent data from mammalian cells also suggest that bringing together PREs, even those located on different chromosomes, may enhance the level of PcG-mediated silencing [34–36]. Such clustering of multiple PcG target sites at a single-gene locus has not been discovered yet in mammalian cells where, unlike in *Drosophila*, PREs are not defined, thus restricting our ability to assay for such functional crosstalk between multiple PcG-bound elements as well as PcG-mediated long-range chromatin interactions. Moreover, how dynamic changes in such PcG-organized chromatin conformation states interplay with other epigenetic modifications, especially DNA methylation, and relate to different degrees of transcriptional repression, is not known.

One model setting to explore the function of PcG concerns genes that, in adult cancer cells and embryonic cells, are marked by PcG and have helped foster the concept that adult cancers arise from abnormal expansion of stem or early progenitor cells [37–39]. The concept is that the repressed state of some important regulatory genes mediated by PcG in ES and perhaps adult stem cells may predispose these genes to a very tight silencing, in combination with aberrant promoter DNA hypermethylation, during adult tumor initiation and progression [38]. These PcG marked genes, while bearing promoter DNA methylation in adult cancers, generally are maintained DNA methylation-free in ES cells, and germ cell carcinomas (embryonic carcinoma; EC) directly derived from embryonic germ cells [38]. In the current study, we have intensively studied one such gene, *GATA-*4*,* as a model gene for how PcG complexes and DNA methylation may maintain reduced transcription of mammalian genes. We present distinct evidences for the participation of long-range interactions, around a single gene, by chromatin looping as a mechanism for PcG-mediated and DNA methylation–dependent gene silencing in mammalian cells, and we show how different states of gene expression, PcG occupancy, and DNA methylation are associated with such loops as a function of stem cell state, lineage commitment of such cells, and complete gene silencing in adult cancer cells.

## Results

### The *GATA-*4 Locus Is Spatially Organized in a Topologically Complex Multi-Loop Conformation in Undifferentiated Tera-2 Cells, Which Is Lost upon Retinoic-Acid–Induced Cellular Differentiation

To begin exploring relationships between PcG occupancy and chromatin conformation of *GATA-*4 in different cell states, we first searched for whether the promoter of this gene has any long-range associations in a well characterized human EC cell line: undifferentiated Tera-2 cells. For this, we used the associated chromosome trap (ACT) assay [22], the results of which provided us with the clues that this locus might be engaged in certain long-range interactions, with a particularly strong link between the proximal promoter region and a site approximately 18.3 kb upstream from the promoter (Figure S1).

**Figure 1:**
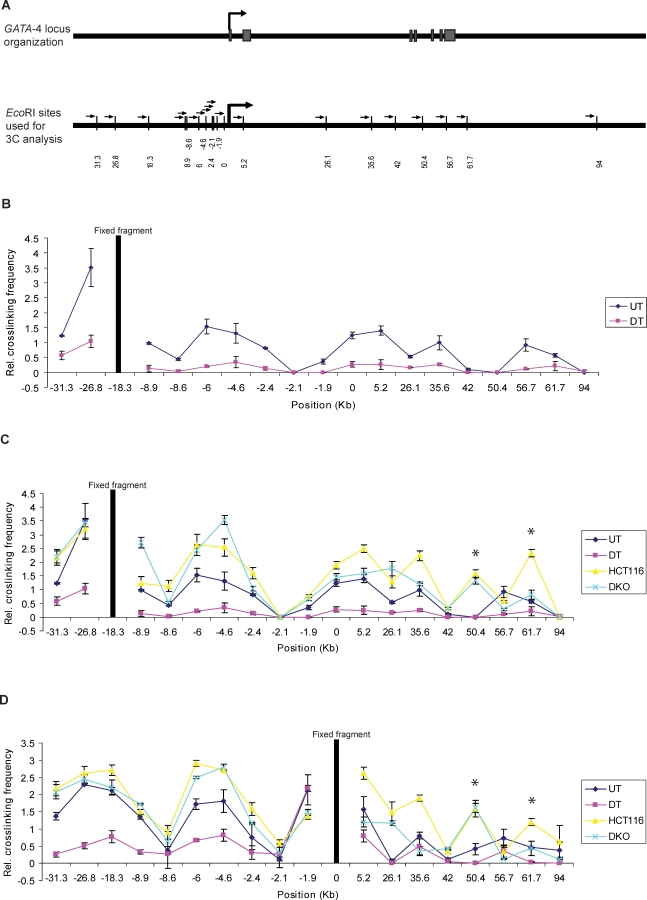
The Long-Range Chromatin Interactions at the *GATA-*4 Locus in Undifferentiated EC Cells, HCT116, and DKO Cells and Its Loss in Differentiated EC Cells, as Mapped by Chromosome Conformation Capture (3C) Assay (A) Representation of the genomic organization of the human *GATA-*4 locus (upper panel) and *Eco*RI sites used for this study (small vertical bars, lower panel), with distance in kb along the *x*-axis (lower panel). The seven exons of the gene are shown as grey boxes, intervened by six introns. The large arrowhead represents the transcription start site. The gene spans approximately 55 kbp on 8p23.1–22. The *Eco*RI site nearest to the transcription start site is 0 on the x-axis. The fragments at −18.3 (B and C) and promoter (0) (D) were used as bait in the 3C assay for bidirectional search of physical partners. The small arrows next to each *Eco*RI site represent the location and the direction of primers used for the 3C assay in Figures 1 and 4. (B) The relative crosslinking frequency of different regions interacting with the −18.3 fragment (fixed fragment, the thick black line) across the *GATA-*4 locus in undifferentiated Tera-2 cells (blue dots) and is compared to EC cells differentiated by ATRA treatment (purple dots) . Standard error of the mean is indicated by the brackets around each dot, which represent the average derived from at least five independent samples. The value of relative crosslinking frequency is plotted on the *y*-axis and the locations of various *Eco*RI sites (in kb) used for the 3C analysis are plotted on the *x*-axis. The calculation of relative crosslinking frequency between two given *GATA-*4 fragments was done essentially as described by others [81], which allows a direct comparison between the different cell types used in the 3C assay by correcting for possible variants. *ERCC*3 fragments separated by approximately 10 kb were used for normalizing crosslinking frequency. UT= undifferentiated Tera-2 cells, DT= differentiated Tera-2 cells. (C) The spatial organization of the *GATA-*4 locus in the four cell types examined: HCT116, DKO, undifferentiated Tera-2, and differentiated Tera-2 cells. The graph is plotted similarly to (B) (for explanation of symbols, refer to (B)). Relative crosslinking frequencies of different regions with the −18.3 fragment in undifferentiated Tera-2 cells (UT), differentiated Tera-2 (DT), HCT116, and DKO cells are shown as blue, purple, yellow, and green dots, respectively, as indicated to the far right of the panel. The two novel interactions discovered in HCT116 cells, located at +50.4 and +61.7 kb, are marked by asterisks, one of which (+61.7 Kb) had reduced crosslinking frequency in DKO cells. (D) The results of 3C analysis in the four cell types using the promoter fragment (0) as bait, instead of the −18.3 fragment. The graph is plotted similarly to (B) (for explanation of symbols, refer to (B)). Relative crosslinking frequencies of different regions with the promoter (0) fragment in undifferentiated Tera-2 cells (UT), differentiated Tera-2 (DT), HCT116, and DKO cells are shown as blue, purple, yellow, and green dots, respectively, as indicated to the far right of the panel.

To discover other sequences that might be part of the chromatin higher order structure at the *GATA-*4 locus, we performed systematic chromosome conformation capture (3C) analysis and started an unbiased 3C walk in this gene locus region extending over 125 kb including approximately 32.5 kb upstream and 92.5 kb downstream of the transcription start site (Figure 1A) using two different fixed (“bait”) fragments, one at −18.3 kb, the site captured in the initial screening ACT assay, and the other one at the promoter (0). We measured the crosslinking frequencies between these baits and the various *Eco*RI sites spread throughout the locus (Figure 1B). We found that, in undifferentiated Tera-2 cells, the −18.3-kb fragment interacts frequently with fragments at −31.3, −26.8 , −8.9, −6, −4.6, +5.2 , +35.6, and +56.7, in addition to the promoter fragment (0) (Figure 1B and 1C). We found almost identical results when we switched the bait and performed similar 3C analysis using the fragment spanning the promoter region as the fixed fragment (Figure 1D). Again, the promoter fragment interacts with the above fragments, in addition to the −18.3-kb fragment, thus supporting the findings. We did note that the frequency of crosslinking differs slightly when the baits are switched. This has also been noticed previously by others for results of 3C analyses done with different baits at the same locus [27,34,40,41]. The differences are probably due to the way chromatin is organized in terms of steric determinants that affect ligation frequency. Nonetheless, the pattern of crosslinking frequency with different chromatin regions across the locus is very similar, even when the baits are switched (correlation analysis; *R* ^2^ = 0.8). Moreover, when we used different primer combinations from the same bait, the crosslinking frequencies were almost identical (correlation analysis; *R* ^2^ > 0.95).

**Figure 2:**
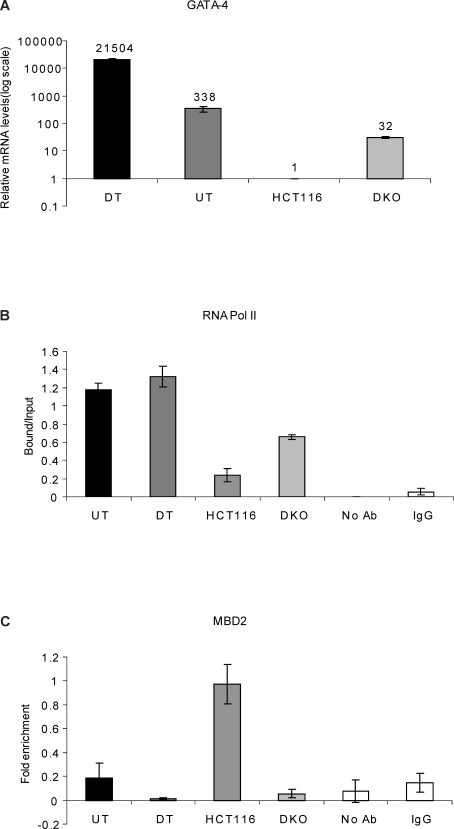
The Frequency of Long-Range Chromatin Interactions at the *GATA-*4 Locus Correlates with the Transcriptional Status of the Gene and Is Associated with the Differential Occupancy of RNA Pol II and MBD2 at the *GATA-*4 Promoter in the Four Cell Types (A) Realtime RT-PCR analysis for relative comparison of *GATA-*4 transcript levels reveals differences among the four cell types- HCT116, DKO, undifferentiated Tera-2 and differentiated Tera-2 cells, which correspond well with the spatial organization of the *GATA-*4 locus. The numbers on top of the bars indicate fold up-regulation in *GATA-*4 mRNA levels in the three cell-types as compared to the HCT116 cells, which was just above a baseline value and is set to 1. Note that the relative mRNA levels on the *y*-axis are plotted logarithmically. (B) ChIP analysis for RNA Polymerase II occupancy at the *GATA-*4 promoter in undifferentiated Tera-2, differentiated Tera-2, HCT116, and DKO cells. ChIP was performed using antibodies against the initiating form of RNA Polymerase II followed by PCR using primers spanning the promoter region and the products were resolved on a 2% agarose gel and quantitated using KODAK Gel Logic 2000 imaging system. Average enrichments, from separate assays, are plotted on the *y*-axis as the ratio of precipitated DNA (bound) to the total input DNA (1:100 dilution) and are compared with the control samples (no antibody and IgG). Standard errors of the mean are indicated by the brackets. UT = undifferentiated Tera-2 cells, DT = differentiated Tera-2 cells. (C) MBD2 is enriched at the *GATA-*4 promoter region only in HCT116 cells and not in the other three cell types. ChIP was performed using antibody against MBD2, and the precipitated DNA was amplified by real-time qPCR using primers specific for the promoter CpG island in UT, DT, HCT116, and DKO cells. Enrichments are presented as folds of total input and are compared with the control samples (no antibody and IgG). Standard errors of the mean are indicated by the brackets. UT= undifferentiated Tera-2 cells, DT= differentiated Tera-2 cells.

To test the correlation between the topologically complex structure observed above with the epigenetic state as well the transcription potential of the *GATA*-4 promoter, we performed similar 3C analyses upon ATRA (all trans-retinoic acid)–induced differentiation of these cells towards a neuronal lineage [38]. In this setting, the *GATA-*4 gene is transcriptionally activated approximately 60-fold from the low basal level in the EC cells (Figure 2A). In striking contrast to the presence of multiple long-range associations among chromatin elements in undifferentiated Tera-2 cells, the ATRA treatment led to a virtually complete loss of these interactions (*p-*value << 0.05 in all cases) and the locus acquires an almost linear conformation (“active conformation”) as indicated by results from the 3C assays using either the fragment at the −18.3-kb position (Figure 1B and 1C) or the fragment that spans the proximal promoter region as bait (Figure 1D). These results indicate that the *GATA-*4 locus is spatially organized in a multi-loop conformation, forming a pre-repressive chromatin hub (pre-RCH) that is specific to the poised state of the gene, in undifferentiated Tera-2 cells, where transcription of the gene is substantively repressed as compared to expression of the gene in a neural, differentiated state.

**Figure 3:**
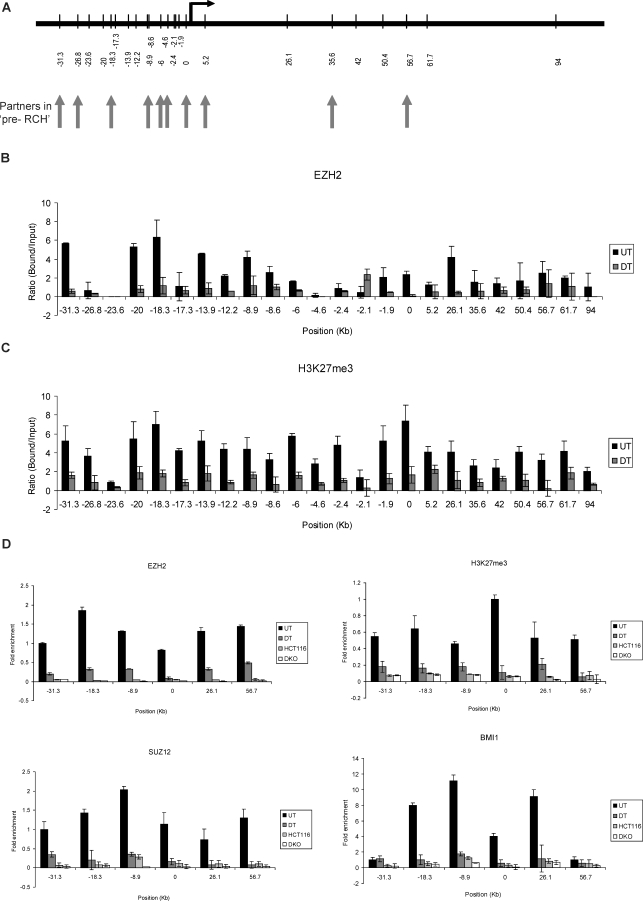
PcG Proteins and Associated H3K27 Marks Occupy Broad Chromatin Domains throughout the *GATA-*4 Locus (A) Schematic representation of location of various chromatin regions (in kb) at the human *GATA-*4 locus, queried by semiquantitative ChIP analysis for various components of the PcG repression system, including all the *Eco*RI enzyme sites used for the 3C analysis. The vertical arrows point towards the *Eco*RI sites that were discovered to be involved in long-range chromatin interactions in undifferentiated Tera-2 cells. (B and C) Quantitation of the gel-based PCR analysis of various fragments at the *GATA*-4 locus for EZH2 (B) and H3K27me3 (C) occupancy for undifferentiated and differentiated Tera-2 cells. Following the PCRs using primers spanning the *Eco*RI sites shown in (A), the products were resolved on a 2% agarose gel and quantitated using the KODAK Gel Logic 2000 imaging system. Average enrichments, from separate assays, are presented as the ratio of precipitated DNA (bound) to the total input DNA (1:100 dilution) and are plotted on the *y*-axis. Standard errors of the mean are indicated by the brackets. Black bars represent undifferentiated Tera-2 cells (UT), and grey bars represent differentiated Tera-2 cells (DT). (D) ChIP was performed using antibodies against EZH2, H3K27me3, SUZ12, and BMI1. The precipitated DNA was amplified by real-time qPCR using primers specific for some selected fragments (−31.3, −18.3, −8.9, 0, +26.1, and +56.7) in undifferentiated Tera-2 cells, differentiated Tera-2 cells, HCT116, and DKO cells. Enrichments are presented as folds of total input.

### The Multi-Loop Organization of the *GATA-*4 Locus Is Essentially Conserved and Further Tightened in Adult Colon Cancer Cells, Where the Promoter CpG Island Is DNA Hypermethylated and the Gene Is Fully Silenced

We next tested whether the *GATA-*4 locus has different or similar spatial organization in the adult colon cancer cell line (HCT116), where the gene promoter is known to be DNA hypermethylated [38,42] and tightly silenced to a degree where transcription is essentially absent as compared with the low expression levels in the undifferentiated Tera-2 cells (Figure 2A). Interestingly, in these non-expressing cells, the pattern for chromatin elements involved in long-range interactions remained very similar to that in the undifferentiated Tera-2 cells. However, with only few exceptions, the intensity of interaction was found to be increased for fragments throughout the locus (2- to 3-fold for some regions) in HCT116 cells as compared to the undifferentiated Tera-2 cells, either using the −18.3-kb fragment (Figure 1C) or the fragment at the promoter region (0) (Figure 1D) as bait for 3C assays. These differences in the intensity of long-range interactions in HCT116 versus undifferentiated Tera-2 cells were statistically highly significant (*p-*value << 0.05 for all regions, except for fragments at −26.8, −8.9 and +56.7). Interestingly, this “super silenced” state of the *GATA-*4 locus in HCT116 cells seems to recruit additional partners to this chromatin hub, as we discovered two novel fragments, located at +50.4 and +61.7 kb (marked by asterisks in Figure 1C and 1D), with high crosslinking frequency with either the −18.3-kb or the promoter (0 kb) fragment bait and which were absent in undifferentiated and differentiated Tera-2 cells (Figure 1C and 1D). It is possible, then, that these additional partners (+50.4 and +61.7) represent binding sites for other repressor proteins and/or are needed to stabilize the repressed chromatin hub when DNA methylation becomes one of the driving forces for repression, in addition to PcG, thus adding an additional layer of silencing ensuring no “leakiness” in *GATA-*4 transcription.

### The GATA-4 Locus Acquires an “Intermediate” Spatial Conformation in Isogenic Colon Cancer Cells Lacking DNA Methylation

We further investigated the spatial organization of the *GATA-*4 locus in isogenic cell lines lacking DNMT1/DNMT3B (DKO). In this setting, as we have previously reported [42], the *GATA-*4 promoter becomes devoid of DNA methylation and the gene is re-expressed, although we now show that the expression is still ∼10-fold lower and ∼ 670-fold lower when compared to undifferentiated and differentiated Tera-2 cells, respectively (Figure 2A). This result suggests that induced expression by DNA demethylation in these cancer cells may occur in an environment that lacks factors for full differentiation and transcriptional activation, perhaps because the retained stem/precursor characteristics of cancer cells may still help keep the gene in a relatively repressed state. This probability is reflected by the fact that, as measured by chromatin immunoprecipitation (ChIP) assay, RNA polymerase is equally highly enriched at the *GATA-*4 promoter in both undifferentiated and differentiated Tera-2 cells, almost completely absent in wild-type HCT 116 cells, and only modestly enriched in DKO cells (Figure 2B).

Importantly, we find from the 3C analysis using either the −18.3-kb fragment (Figure 1C) or the fragment spanning the promoter (0) (Figure 1D) as bait that, in the DKO cells, while the frequency of long-range interactions are modestly affected in the far 5′ region, they are significantly decreased more toward the 3′ regions. These latter 3′ differences in crosslinking frequency for HCT116 versus DKO cells are significant at a *p-*value << 0.05 for the fragments located at −2.4, +5.2, +35.6, +50.4, and +61.7 (using either bait), at the promoter (0) fragment (using −18.3 bait) and at the −18.3-kb fragment (using promoter fragment as bait) and the frequency of interaction with these fragments now approaches the levels seen in the undifferentiated Tera-2 cells (Figure 1C and 1D).

To rule out that the 3C results obtained with the four cell types do not simply reflect cell-type–specific differences in the efficiency of some step in the 3C protocol, we tested *ERCC*3 as a control locus that is not a PcG target and not subject to DNA hypermethylation. Using the 3C methodology, we analyzed interaction between a constant fragment and four other fragments distributed throughout the *ERCC*3 locus (Figure S2). The results revealed that this locus acquires a similar conformation in all four cell types, with no noticeable differences with respect to the chromatin long-range interactions at the locus. In addition, to ensure that our 3C data does not reflect differences in formaldehyde “crosslinkability” (which would affect restriction digestion efficiency and ultimately 3C results) due to the altered biochemical properties of the locus, we performed a simple PCR-based assay for analyzing restriction digestion efficiency as done previously by others [23,25,40,43,44] . In this control, PCRs done with primers spanning various enzyme sites involved in this study confirmed that all of them were efficiently digested to similar extent in all the four cell types under investigation (Figure S3A). These additional experiments led us to strongly believe that the differences observed at the *GATA*-4 locus are a result of specific changes in chromatin conformation between each of the cell types under investigation.

In effect, then, the above differences in the DKO versus the wild-type HCT116 cells may represent a return of the chromatin conformation of the gene toward the poised transcription state similar to that seen in the undifferentiated EC cells. The data suggest, in this regard, that these slight differences in higher order chromatin organization of the *GATA*-4 locus between HCT116 and the DKO cells where we observed a slight “loosening” in the spatial organization might have important ramifications for *GATA-*4 transcription. The conformation could allow modest access, in DKO cells, of the basal transcriptional machinery and/or regional enhancers to the promoter, resulting in return of Pol II (Figure 2B) and distinct, but minimal, transcription activation (Figure 2A). This increase in gene expression is also similar to the transcription levels achieved when many DNA hypermethylated and silenced genes are re-expressed in the setting of DNA demethylation in adult cancer cells, including DKO cells [45,46]. The data also provide a novel look at how DNA methylation may contribute directly to the higher order spatial organization of a particular locus in cancer cells.

### EZH2 and Associated H3K27me3 Marks Blanket the *GATA-*4 Locus in Undifferentiated Tera-2 Cells

Having obtained evidence for a topologically complex looping conformation surrounding the *GATA-*4 locus when the gene is in a low or completely repressed transcription state, we could now return to the question of PcG participation in this process. All of the data presented to this point, and previous observations that PcG proteins are an integral part of the *GATA-*4 silencing machinery at the proximal promoter in undifferentiated Tera-2 cells in addition to colon cancer cells [38,45,46], now prompted us to investigate PcG occupancy throughout the locus. To get an idea of the extent to which PcG heterochromatin marks are spread around the promoter, we turned our attention to recent data in our lab [46] wherein, using ChIP-chip assays [47] with Agilent gene promoter tiling arrays, we have mapped several histone modifications genomewide including the PcG mark H3K27me3 in HCT116 cells and focused specifically on the 64 probes across the *GATA-4* locus spanning the transcription start site (−5428 to +6537). The findings with this fine mapping of PcG inactive marks provided us with the clues that PcG marks are not simply restricted to a region near the transcription start site of *GATA-*4 gene, but rather have a broader distribution, with slightly higher enrichments towards the 5′ region (Figure S4). This prompted us to expand our study to cover the entire *GATA*-4 locus in the four cell types under investigation.

We performed ChIP assays using antibodies against EZH2, H3K27me3, and other PcG constituents followed by semiquantitative gel-based detection, and reconfirmation by real-time PCR. As shown in Figure 3B–3D, the key PcG proteins, EZH2, SUZ12, and BMI1, all have very strong enrichment at several fragments far upstream and at some fragments far downstream of the promoter region in undifferentiated Tera-2 cells. Almost similar, though importantly not completely overlapping, patterns were obtained for H3K27me3 marks, which appear to have the best correlation to the frequency of long-range interactions involving those fragments previously outlined in Figure 1. Thus, there were sites where little or no EZH2 was enriched but higher levels of H3K27me3 marks were observed (−26.8, −17.3, −6, −4.6, −2.4, +5.2) (Figure 3B and 3C). We also performed ChIP analysis using an H3K4me2-specific antibody and discovered H3K4me2 enrichment around the proximal promoter region, simultaneously with the broader enrichment of H3K27me3 (Figure S5). Importantly, this mark is retained, while H3K27me3 is lost when the cells are induced to differentiate with retinoic acid (Figure S5), consistent with resolution of the bivalent chromatin to active chromatin. The pattern for these marks is fully consistent with the presence of a bivalent chromatin state that marks the *GATA*-4 promoter as “poised” in a ES cell-like state until the gene is activated by differentiation-specific signals such as those induced by ATRA [38]. Interestingly, in Tera-2 cells, as for the long-range interactions demonstrated previously, the occupancy of EZH2, other PcG components (SUZ12, BMI1), in addition to the H3K27me3 marks was severely reduced at all the sites assayed when these cells were directed to differentiate using ATRA and the gene is robustly activated (Figure 3B–3D). These results indicate that EZH2, other PcG proteins, and associated H3K27me3 marks occupy large chromatin domains around the entire *GATA-*4 locus, and the complete PcG occupancy pattern is lost with induced differentiation.

We also performed real-time quantitative PCR analysis to study enrichments of PcG proteins and H3K27me3 marks in HCT116 wild-type and DKO cells. Interestingly, despite the generally higher frequency of many of the long-range interactions in these cells (Figure 1C and 1D) and higher levels of expression of PcG components such as EZH2 in colon cancer cells (HCT116 and DKO) than in any of the EC cells (Figure S6), the levels of the above PcG components and the associated H3K27me3 marks were found to be lower at all fragments in HCT116 and DKO cells as compared to undifferentiated Tera-2 cells (Figure 3D) (also compare Figure 3B and 3C with Figure S7A). Note, however, that although diminished on the graph relative to the levels in the Tera-2 cells, clear enrichments of PcG proteins such as EZH2 and its associated mark H3K27me3 were seen across the regions being examined in HCT116 and DKO cells as compared to the control samples and various fragments at the CDKN2B locus that are not enriched for these marks in these cell types [46] and act as negative controls (Figure S7).

### EZH2 Knockdown Affects Long-Range Interactions at the GATA-4 Locus and GATA-4 Transcription

**Figure 4:**
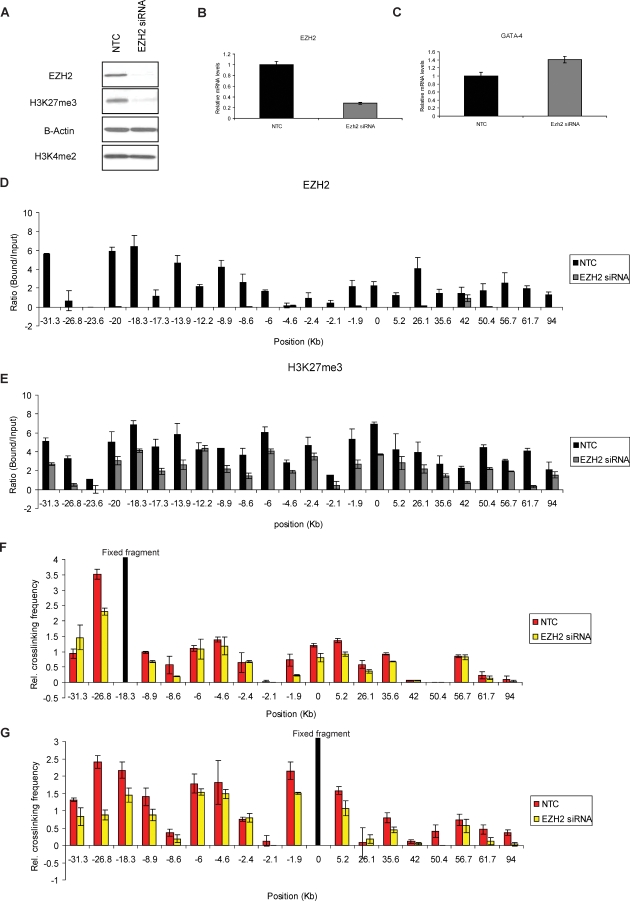
siRNA-Mediated Knockdown of EZH2 in Undifferentiated Tera-2 Cells Impairs Long-Range Associations at the *GATA-*4 Locus with Marginal Effect on *GATA-*4 Transcription (A) Effects of the siRNA knockdown of EZH2 on EZH2 protein and the corresponding histone mark H3K27me3. For control assays, a random-sequence siRNA (NTC) was transfected in parallel. The overall levels of the active mark, H3K4me2, remain unaffected indicating that the targeting is specific for EZH2 and the subsequent effects on the H3K27me3 mark. (B) Effects of the siRNA knockdown on EZH2 mRNA levels and, in (C), on *GATA-*4 transcript levels. Transcription of *GATA-*4 is slightly increased in siRNA treated cells as compared to NTC (C). (D and E) Following ChIP assay, quantitation of the gel-based PCR analysis of various fragments at the *GATA*-4 locus was performed for EZH2 (D) and H3K27me3 (E) occupancy for NTC versus EZH2 siRNA treated Tera-2 cells. Following the PCRs using primers spanning *Eco*RI sites shown in Figure 3A, the products were resolved on a 2% agarose gel and quantitated using the KODAK Gel Logic 2000 imaging system. Average enrichments, from separate assays, are presented as the ratio of precipitated DNA (bound) to the total input DNA (1:100 dilution) and are plotted on the *y*-axis. Standard errors of the mean are indicated by the brackets. Black bars represent NTC and grey bars represent EZH2 siRNA treated Tera-2 cells. (F and G) 3C analysis using either the −18.3 fragment (F) or the promoter fragment (G) as bait indicates a noticeable reduction in the relative crosslinking frequency of certain regions in EZH2 siRNA treated cells as compared to NTC. The graph is plotted exactly as in Figure 1B with similar symbols.

The above ChIP data strongly raise the possibility, when combined with our looping observations, that PcG complexes, and especially the PcG-mediated repressive histone modification marks (H3K27me3), might, through being brought in close physical proximity by chromatin looping, cooperate to mediate degrees of transcriptional repression for an individual gene. Therefore, we next tested whether PcG components might directly facilitate organization of the chromatin superstructure at the *GATA*-4 locus by mediating long-range chromatin interactions, which, in turn, contribute to *GATA*-4 repression. Toward this end, we used RNA interference (RNAi) methodology and examined the relationship of EZH2 and H3K27me3 enrichment throughout the *GATA*-4 locus with the spatial organization of the *GATA-*4 locus as well as *GATA-*4 expression in undifferentiated Tera-2 cells. We transiently transfected Tera-2 cells with siRNA specific for EZH2 or a non-targeting control (NTC). Both Western blot and real-time RT-PCR confirmed efficient knockdown of approximately 70%, of both overall cellular levels of EZH2 and the corresponding H3K27me3 mark (Figure 4A and 4B). We also queried, by ChIP assay, the local enrichments of EZH2 and H3K27me3, after such EZH2 depletion. We found that the EZH2 protein was non-detectable at most of the chromatin regions being analyzed after siRNA-mediated knockdown of EZH2 (Figure 4D). Importantly, however, although the H3K27me3 mark showed varying degrees of depletion at virtually each site examined, the loss was much less prominent than for EZH2 (Figure 4E).This was not surprising, because there is ample precedent for H3K27me3 marks to be much more stable, than presence of the enzyme (EZH2) that catalyzes it [48–51].

Interestingly, with such virtual depletion of EZH2 and less severe reduction of H3K27me3 in cells treated with EZH2 siRNA, the 3C analysis, done either with the −18.3-kb fragment or the promoter (0) region as bait, revealed that the frequency of long-range interactions at various sites was reduced to different degrees as compared to the NTC (Figure 4F and 4G). Similar levels of reductions were also seen in a recent *Drosophila* study upon knockdown of Polycomb components [34]. Most importantly, the degree of local loss of H3K27me3 marks at various chromatin regions of the *GATA*-4 locus correlated better with the degree of reduction in the frequency of long-range chromatin interactions involving those sites (correlation analysis; *R* ^2^ = 0.7 , *p*-value = 0.002172), than the protein EZH2 itself. The levels of diminution in the frequency of long-range associations was also statistically highly significant at a *p-*value of << 0.05 for fragments at −26.8, −8.9, −1.9, 5.2, and 35.6, while using either bait for the 3C analysis. The differences were also similarly significant for fragments at −8.6, 0, 26.1 (only with the −18.3 fragment as bait) and −31.3, −18.3, 50.4, 61.7, 94 (only with the promoter fragment as bait). Together, these results suggest that EZH2, and most strongly, its catalyzed mark H3K27me3, may physically play a role in the higher order chromatin organization observed at the *GATA*-4 locus in undifferentiated Tera-2 cells.

Such reduction in the frequency of long-range interactions upon siRNA-mediated depletion of EZH2 and the associated H3K27me3 marks correlated with a modest, but distinct (1.5-fold), increase in *GATA-*4 transcription (Figure 4C). Interestingly, as discussed earlier, *GATA-*4 is transcriptionally activated approximately 60-fold from the low basal level in undifferentiated Tera-2 cells upon receipt of differentiation cues, which also parallels severe down-regulation of PcG components and associated H3K27me3 marks globally as well as locally at target promoters, including *GATA*-4 (Figures 2A and 3B–3D and Figure S6)[38]. These observations are also in agreement with our recent findings that, for ten different target genes, the loss of EZH2 in Tera-2 cells needs to be accompanied by the presence of differentiation-specific signals such as those induced by ATRA to achieve robust transcriptional up-regulation [36].

### Multiple CpG Islands throughout the *GATA-*4 Locus Are DNA Methylated in HCT116 Cells but Are Maintained in a Methylation-Free State in Tera-2 Cells

**Figure 5:**
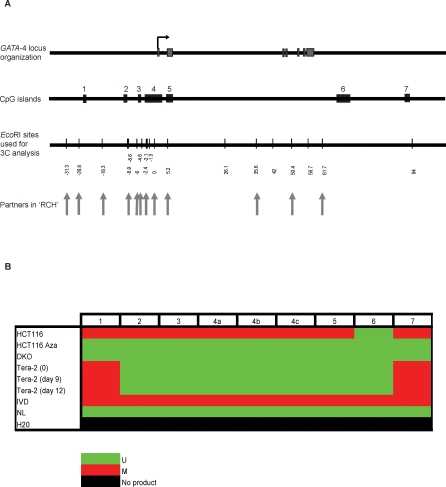
Multiple CpG Islands Spread along the *GATA-*4 Locus Are Methylated in HCT116 Cells but Are Unmethylated in Undifferentiated Tera-2 Cells, Differentiated Tera-2 Cells, HCT116 Cells Treated with the Demethylating Agent 5-Aza-2′deoxycytidine, and DKO Cells (A) The uppermost panel depicts the genomic organization of the human *GATA-*4 locus as in Figure 1A.The drawing below it shows the location of seven CpG islands used for MSP analysis with reference to the *GATA-*4 locus map shown above it. The third panel is a schematic representation of various *Eco*RI sites (thin bars) used in the 3C analysis, and the vertical arrows point toward the *Eco*RI sites that were discovered to be involved in long-range chromatin interactions in HCT116 cells. (B) The MSP results for all the 7 CpG islands are shown (island 1–7) for the four cell lines. CpG island 4 spanning the transcription start site, being very big, was split in three parts, 4a, 4b, and 4c for analysis. In vitro methylated DNA (IVD) and normal human peripheral lymphocytes (NL) served as the positive and negative methylation controls, respectively. The CpG islands 2 to 5 were methylated only in HCT116 cells but were unmethylated in undifferentiated Tera-2, differentiated Tera-2 (both day 9 and day 12 of ATRA induced differentiation), HCT116 cells treated with 5-Aza-2′deoxycytidine and DKO cells while CpG island 6 was always unmethylated in each of these cases. CpG islands 1 and 7 were methylated in HCT116, undifferentiated Tera-2 and differentiated Tera-2 cells but were unmethylated in HCT116 cells treated with 5-Aza-2′deoxycytidine and DKO cells. U = unmethylated (green color); M= fully methylated (red color); No product = H2O control (black color).

The *GATA-*4 gene is frequently silenced in association with hypermethylation of a CpG island surrounding the transcription start site in a variety of tumors including colon, gastric, lung, and ovarian, as summarized in Ohm et al [38]. Our discovery of long-range chromatin interactions prompted us to search more precisely for the location of CpG islands within the region that may potentially be brought together by chromatin looping and might contribute to silencing in some way. Bioinformatics analysis revealed seven CpG islands scattered throughout the 125-kb region encompassing the gene and the region analyzed for chromatin conformation (Figure 5A). We performed methylation-specific PCRs (MSPs) to study the methylation status of all of these CpG islands in undifferentiated Tera-2, differentiated Tera-2 (day 9 and day 12 of differentiation upon ATRA treatment of the cells), HCT116, HCT116 cells treated with the demethylating agent 5-Aza-2′deoxycytidine, and DKO cells (Figure 5B).

All of these CpG islands, including the CpG island 4 which spans the transcription start site of *GATA*-4, were methylated in HCT116 cells, with the exception of CpG island 6. The methylation was lost in each of the islands when the cells were treated with 5-Aza-2′deoxycytidine and in the DKO cells lacking DNMT1/DNMT3B. Interestingly, these CpG islands were unmethylated in undifferentiated and differentiated Tera-2 cells with the exceptions of CpG islands 1 and 7, found at the most 5' and 3' regions respectively, which were methylated. It is tempting to speculate that the clustering of multiple methylated CpG islands by chromatin looping in HCT116 cells might serve a similar function to, or augment the clustering function of, PcG occupied sites, to maximally transcriptionally repress *GATA-*4*.* This, in essence, may switch the repression to be more DNA methylation–dependent instead of being PcG-dependent [1,52–54], and our finding of definite PcG marking in HCT116 cells, but at distinctly lower levels than in the EC cells, may fit with this hypothesis.

If DNA methylation of multiple CpG islands indeed contributes to the increased frequency of long-range interactions, and the increased gene silencing that we observed for the *GATA*-4 locus in HCT116 cells, what other factors might be involved? Importantly, we find that the methyl cytosine binding protein MBD2 is enriched at the *GATA-*4 promoter CpG island (CpG island 4) only in HCT116 cells and not in the other three cell types under study (Figure 2C). Similar MBD2 occupancy also occurs at each of the other methylated CpG islands in HCT116 cells (Figure S8). In DKO cells, loss of DNA methylation at each of these CpG islands leads to loss of MBD2 enrichments (Figure S8), which accompanies a decrease in the frequency of certain long-range interactions (Figure 1C and 1D). However, there is increased expression of EZH2 (Figure S6) and higher enrichments for PcG/H3K27me3 at the *GATA*-4 locus in DKO cells relative to HCT116 cells (Figure S7A). This may provide some explanation for the retained loops in DKO cells although we cannot rule out that other mechanisms could additionally contribute to the maintained high frequency of certain long-range interactions in these cells.

## Discussion

**Figure 6:**
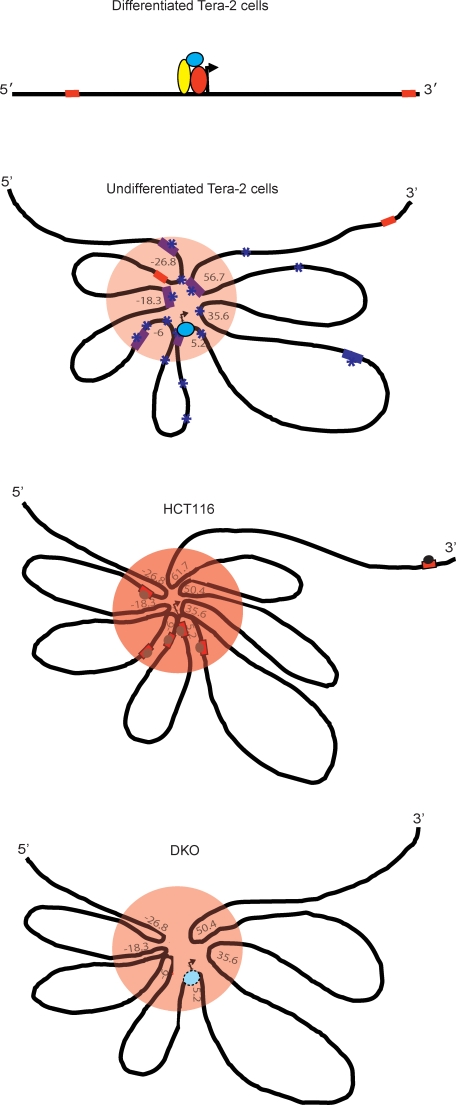
A Model for How the Spatial Chromatin Conformations Observed May Be Visualized around the *GATA-*4 Locus and Correlated with the Different Transcription States Seen in the Cells Studied In the second panel (undifferentiated Tera-2 cells), the PcG-dependent long-range interactions, creating a pre-RCH (orange circle area) at the *GATA*-4 locus, are shown. This hub is associated with a poised transcription state, in which RNA Pol II is present at the promoter (sky blue oval), and low transcriptional activity. Purple squares and asterisks indicate regions showing strong enrichments for PcG proteins and repressive H3K27me3 marks respectively. Only the far upstream and most downstream CpG islands (CpG islands number 1 and 7 in Figure 5) are DNA methylated (red squares). This chromatin higher order structure is plastic in nature in the sense that the repressive hub can be dissolved when the cells are induced to high transcription of *GATA*-4 by differentiation cues in ATRA treatment of EC cells (differentiated Tera-2 cells, top panel). In this active state, PcG occupancy and H3K27me3 enrichment are severely diminished throughout the *GATA-*4 locus and the locus acquires an almost linear conformation. Such state of high transcription involves recruitment of other transcriptional coactivators (other colored ovals) to the RNA Pol II bound proximal promoter region around the transcription start site (indicated by the arrow) which are now made available and have access to the region in differentiated Tera-2 cells. The frequency of long-range interactions are depicted as increased, associated with DNA methylation of multiple CpG islands across a wide upstream region (red squares within the orange oval), forming a much tighter RCH in the HCT116 adult cancer cells (third panel). This state is accompanied by RNA Pol II exclusion and MBD2 recruitment (grey oval) at methylated CpG islands including the ones near the gene promoter. The result is severe repression of *GATA-*4 transcription to a fully silenced state. The multiple methylated MBD2-bound CpG islands are depicted as serving the same function as, or augmenting the effects of, the multiple PcG occupied and H3K27me3 enriched sites seen in undifferentiated Tera-2 cells. The bottommost panel shows the spatial organization of the locus for the state of *GATA*-4 in the isogenic cancer cell line of HCT116 (DKO) where knockout of DNA methyltransferases (DNMT1A/3B) (DKO) has induced overall depletion of DNA methylation. The long-range interactions in these cells are slightly destabilized, with decrease in the frequency of long-range chromatin interactions at several downstream fragments at the *GATA-*4 locus, including the promoter. The result is a higher order chromatin conformation state intermediate to that in the wild-type HCT116 cells (RCH) (above panel) and that in the undifferentiated Tera-2 cells (pre-RCH) (second from the top panel). The consequence is a modest enrichment of RNA Pol II at the promoter and return of the *GATA*-4 transcription to a more poised state with a low, but definite, level of expression.

The looping model for control of gene expression postulates that various regulatory elements communicate, through direct interactions of chromatin constituents, such that certain regions are excluded from the loop [26]. Several reports have shown the existence of chromatin hub–like structures specific to the active state of a gene, and the term “active chromatin hub” (ACH) has been proposed [23,26,27]. In this study, we demonstrate that distant chromatin elements at the *GATA-*4 locus interact to form what might be envisioned as a “pre-repressive chromatin hub” (pre-RCH) in undifferentiated Tera-2 cells where the gene is poised, with presence of RNA Pol II at the start site, in a low to medium expression state (Figure 6). Intriguingly, such a hub, characterized by PcG proteins and the H3K27me3 mark at loop intersection points, also appears to enclose the body of the gene, which may restrict the access of transcription machinery and/or regional enhancers to the promoter [25] and could also be important for other parts of the expression system, such as transcriptional elongation and regulation of other transcription start sites within the gene, which are commonly present as shown in the recent ENCODE study [55,56]*.*

Important to this repressive hub concept and our description that such a hub could be composed of PcG/H3K27me3-enriched chromatin elements spread over 100 kb around a single gene locus, is the possibility that these several PcG interacting regions emanate from other chromatin regions distal to the proximal promoter that act as a recruitment centre for these proteins. One such fragment is located at −18.3 kb, which interacts with a high frequency with the promoter fragment (Figure 1B and 1D) and also shows very strong enrichments for all PcG proteins we assayed including EZH2, BMI1, and SUZ12, as well as the associated repressive mark H3K27me3 (Figure 3B–3D). The PcG apparatus could then spread toward the promoter by looping or linking mechanisms [57] to establish transcriptional silencing (Figure S9). Overall, such an increase in PcG-dependent repressive activity in the vicinity of the promoter leads to an efficient but “plastic” transcriptional down-regulation in embryonic cells that can be reverted to an active conformation by signal transduction signals. Thus, as we now show, this hub-like structure is almost completely abolished when Tera-2 cells are directed to differentiate and *GATA-*4 transcription is robustly up-regulated (Figure 1B and 1D). Our observations also extend previous suggestions in *Drosophila* to mammals, raising the possibility that DNA bound PcG complexes associate with each other and this may lead to strong cooperation between them [34,35,58–60]. In addition, we provide a novel suggestion in mammalian cells that the clustering of multiple PcG target sites spread throughout a single gene locus may enhance the level of PcG-mediated transcriptional silencing of the associated gene. Interestingly, such accumulation of PcG proteins in the vicinity of a single gene promoter and encompassing regions might represent one type of the “PcG body”–like structures, which are thought to be the centers for simultaneous silencing by PcG of multiple target genes [61–65].

All of our data in the present work suggest that, in each of the cell types we have studied, a major factor in maintaining the repressive chromatin superstructure at the gene locus we studied is the PcG machinery in general, and levels of H3K27me3 in particular, which reside at the loop intersection points. The evidence is at least 2-fold. First, in the differentiated EC cells, the loss of long-range interactions is accompanied by a profound loss of PcG constituents, including H3K27me3. Second, the knockdown of EZH2 in undifferentiated EC cells results in a locus-wide diminution in the frequency of long-range interactions, for which the degree best correlates with H3K27me3 enrichment levels. These observations re-emphasize the findings that PcG proteins might accomplish their repressive function by organization of higher order chromatin domains in the genome, the constitution of which are modulated in a development and differentiation-dependent fashion [34,36].

Our present data are particularly interesting with respect to the formation of an RCH in adult cancer cells and its relation to a pre-RCH–like chromatin organization in stem cell–like cells. Our present observations markedly extend our previous work in ES and EC cells [38], and we now additionally provide a novel higher order chromatin conformation link between stem/precursor cells, PcG, and silencing for genes that are DNA hypermethylated in adult cancers. DNA hypermethylation, particularly in CpG islands spanning gene start sites, has now been associated with abnormal transcriptional silencing of many genes in cancers of all types [38,52,66]. Our model would be that some genes with a PcG-mediated poised and plastic silenced state in embryonic or adult precursor cells can be converted, as a function of tumor initiation or progression, into a cancer-specific, DNA methylation–based, stable silencing state in adult cancer cells (Figure 6). Concerning this possibility, the levels of PcG proteins and the associated histone mark, H3K27me3, are higher in EC cells as compared to colon cancer cells (Figure 3D) while the opposite is seen for DNA methylation and a factor that interprets this methylation for transcriptional repression, MBD2 (Figures 2C and 5B and Figure S8). This is further supported by our previous observations that siRNA-mediated knockdown of EZH2 can lead to increased expression of genes when they lack DNA methylation in cancer cells but not for the same genes when they are DNA hypermethylated in cancer cells [67]. Importantly, undifferentiated Tera-2 cells and DKO cells lack DNA methylation of multiple CpG islands spread across the locus, including the one spanning the transcription start, and both have similar spatial organization of the *GATA-*4 locus. Interestingly, despite lack of DNA methylation in DKO cells, certain long-range contacts are only either modestly affected or unaffected. There is concomitantly retention of, and even increases in, enrichment of PcG components and H3K27me3 marks in these regions (Figure S7A). Similar observations were also made for increases of H3K27me3 marks in DKO cells, as assessed by local ChIP, and most recently by ChIP-chip tiling arrays, for the promoter regions of virtually all verified DNA hypermethylated genes including *GATA-*4*, GATA-*5*, SFRP*2*,* and *SFRP*5 [45,46]. Overall cellular levels of EZH2 are also somewhat higher in DKO than HCT116 cells (Figure S6). Thus, although we do not yet completely understand the factors and the underlying mechanisms that could contribute to the higher frequency of certain long-range interactions in DKO cells, we speculate that the PcG proteins, and especially the H3K27me3 mark, in cooperation with other epigenetic repressors, partially preserve the chromatin higher order structures that still keep the gene in a relatively repressed state in the DKO cells (Figure 6).

We now introduce the novel possibility that clustering of multiple abnormally methylated CpG islands, spread throughout a gene locus, may contribute to the increased frequency of long-range chromatin interactions in adult cancer cells. In essence, this interaction might accomplish, add to, or even replace the same function subserved by the clustering of multiple PcG occupied sites, such as found in undifferentiated Tera-2 cells. This replacement might involve recruiting more repressor molecules, such as methyl-cytosine binding protein (MBD) complexes [53,54,68] to the vicinity of genes like *GATA-*4 (Figure 2C and Figure S8)*.* This scenario would then ensure absolute transcriptional silencing. Interestingly, large chromosome regions of abnormal silencing of multiple genes, with interspersed areas of DNA methylated and nonmethylated CpG island regions, have been described in colon cancer cells [69], but not just for a region surrounding a single gene. The ramifications for chromatin spatial conformation of such chromosomal regions in cancer cells have not been explored. However, for the imprinted *Igf2*-*H19* locus, it has already been shown that differentially DNA methylated regions cooperate with each other by physical interaction and partition the locus into distinct chromatin loops to establish proper (imprinted) expression patterns [25,70]. The resulting loop places the *Igf2* promoter in a (hypothetical) silent compartment, inaccessible to the downstream enhancers, thereby not allowing *Igf2* transcription. Our observations suggest that similar dynamics could occur at a nonimprinted locus.

From a basic standpoint, our findings comparing undifferentiated Tera-2, differentiated Tera-2, HCT116, and DKO cells address the issue of direct relationship between spatial organization of an individual gene locus with respect to the chromatin status (PcG occupancy/CpG methylation) as well as different transcription states of the gene (Figure 6). For our study, the level of silencing of a gene seems to be decided by the intensity of interactions between chromatin regions involved in long-range contacts surrounding the gene locus: the higher the frequency, the tighter the silencing of the gene (Figure 6). Our findings raise the issue that while genome-wide DNA methylation studies and tiling studies of individual chromatin marks are providing important clues to gene activity states [11,17,47,71–78], such studies essentially map only linear, rather than three-dimensional chromatin patterns. There is, thus, a need to map chromatin higher order structures, such as the ones we herein describe, across the genome. This will allow appreciation, to the fullest extent, of how these marks are mediating functional aspects of gene expression in terms of key chromatin marks as well as the DNA methylation status of the accompanying CpG islands. Relationships between cell states, such as for cell lineages during development, differentiation transitions in adult cell renewal systems, relationships between stem/precursor cells and cancer cells, may only become fully apparent when position of chromatin marks serve as landmarks for focusing studies upon the chromatin spatial domains they mediate.

From a translational standpoint, these initial systematic studies of the higher-order chromatin structure in a gene frequently silenced by epigenetic mechanisms in multiple cancer types raises an important issue with respect to the hope that targeting reversal of abnormal promoter CpG island DNA demethylation, and resultant re-expression of the abnormal silencing of associated genes, presents a prevention/treatment strategy for cancer [52,79,80]. The comparison of the linear spatial conformation of *GATA-*4 when robustly expressed in ATRA-induced differentiation of EC cells to the retained looped structures of this minimally re-expressed gene when it is DNA de-methylated in DKO cells suggests that more than just the DNA demethylation strategy may be needed. It may be that, without simultaneously restoring missing key signal transduction differentiation cues to pre-neoplastic or frankly neoplastic cells, the full gene expression required for optimal prevention/therapeutic effects will not be achieved. It will be interesting, in this regard, to see whether similar principles of chromatin spatial conformation as we have shown for *GATA-*4 in the present study, apply to other genes frequently silenced by epigenetic mechanisms, and particularly to the large numbers of genes that are abnormally DNA hypermethylated in essentially all primary tumor types [38,66,80].

## Materials and Methods

### Cell culture.

Tera-2, HCT116, and DKO cells were maintained as previously described [38,45]. The colon cancer cell line HCT116 cells were treated with 2μM of 5-aza-2′-azadeoxycitidine or DMSO (Sigma-Aldrich) for 96 h. Fresh media containing 5-aza-2′-azadeoxycitidine or DMSO was added every 24 h.

### ACT and 3C assay.

The ACT assay was done essentially as described by Ling et al. [22].The primers used for PCR and adapter sequence used in the assay are available on request.

The 3C assay was done essentially as discussed by Tolhuis et al. [81] with minor modifications for human cells. Briefly, approximately 10^6^ cells were crosslinked with formaldehyde (Sigma) at a final concentration of 2% at room temperature for 10 min, followed by quenching of the reaction by addition of 0.125 M glycine. Subsequently, the nuclei were prepared by lysing the cells in ice-cold lysis buffer (10 mM Tris pH 8.0, 10 mM NaCl, 0.2% NP-40) containing complete protease inhibitors (Roche). Nuclei were resuspended in the *Eco*RI restriction buffer (NEB) and SDS was added to 0.3% and incubated for 1 h at 37 °C while gentle shaking. Triton X-100 was added to 1.8%, and the nuclei were further incubated for 1 h at 37 °C to sequester the SDS. The crosslinked chromatin was digested overnight at 37 °C with the *Eco*RI restriction enzyme (NEB) while shaking. Next day, the restriction enzyme was inactivated by the addition of SDS to 1.6% and incubation at 65°C for 25 min. The chromatin was heavily diluted (to approximately 2.5 ng/μl) with 1.15 X ligase buffer (NEB), and Triton X-100 was added to 1% and incubated for 1 h at 37 °C with occasional shaking. The DNA was ligated using T4 ligase (NEB) for 4–4.5 h at 16 °C followed by 30 min at room temperature. Subsequently, for reversal of crosslinks, Proteinase K was added to the samples and incubated overnight at 65 °C. Next day, the samples were incubated for 30 to 45 min at 37 °C with RNase, and the DNA was purified by phenol-chloroform extraction followed by ethanol precipitation and finally dissolved in 150 μl of 10 mM Tris pH 8.0 and transferred to 4 °C for short-term or −20 °C for long-term storage.

The PCR analysis was optimized by digesting and ligating a BAC clone, which encompassed the entire *GATA-*4 locus. A novel control was designed and tested for the human *ERCC*3 locus in order to be able to normalize crosslinking and ligation frequency among various cell lines analyzed. The primer sequences and amplification conditions used in the 3C assay are available on request. The *p-*values were calculated by using Student's *t-*test, and the number of observations was at least five for each data point.

The linear range of amplification was determined for all 3C samples by serial dilution and the chosen dilution from the linear range was used for the experiments. PCR products were run on 2% agarose gels and quantified using the KODAK Gel Logic 2000 imaging system. All data points were generated from an average of five different experiments performed in duplicates. Crosslinking frequencies were calculated using the equation described previously [81].

### RNA purification and real-time RT-PCR analysis.

RNA was isolated with the RNeasy kit (Qiagen) according to the manufacturer's instructions. For qPCR analysis, 1 μg of total RNA was reverse transcribed using the Superscript First-Strand Synthesis System (Invitrogen). Quantitative real-time RT-PCR using was performed using QuantiTect SYBER Green PCR Kit (Qiagen). PCR primers and amplification conditions are published previously [38].

### ChIP.

The chromatin was cross-linked with formaldehyde to a final concentration of 1% at room temperature for 10 min. Subsequently, the reaction was quenched by addition of glycine to 0.l25 M. The cells were mildly trypsinized, scraped and transferred to a falcon tube. For each ChIP assay, ∼2 × 10^6^ cells were used. The cell pellets were resuspended in SDS lysis buffer (Upstate Biotechnology) plus complete protease inhibitors, and sonicated to shear the DNA to fragments ranging in size from ∼200–1,000 bp. After removing 50 μl to serve as the “input,” the supernatant was divided equally for each immunoprecipitation. Each lysate was then diluted 10-fold in ChIP dilution buffer (Upstate Biotechnology) and precleared with previously blocked Dynal Magnetic beads purchased from Invitrogen ((Protein A beads (100–02D) and Protein G beads (100–04D)) for 2 h at 4 °C with agitation. The soluble chromatin fraction was collected and either no antibody, IgG, or antibody against the protein or histone modification mark of interest was added and incubated overnight at 4 °C with overhead rotation. Immune complexes were collected for 3 h at 4 °C with agitation using previously blocked Dynal Magnetic beads. The beads and associated immune complexes were washed four times with Low Salt Immune Complex Wash Buffer and once with High Salt Immune Complex Wash Buffer (Upstate Biotechnology). The immune complexes were eluted with freshly made elution buffer (1% SDS, 0.1 mol/l NaHCO_3_) at 65 °C for 30 min, and treatments with proteinase K (500 μg/ml) and RNase A (0.02 μg/ml) were done simultaneously. The cross-links were reversed overnight at 65 °C and DNA was purified by QIAquick PCR Purification Kit (Qiagen) and eluted in total 100 μl of 10 mM Tris pH 8.0. For final PCRs, either 2 μl of undiluted IP or 1:100 diluted input was used. Trimethylated H3K27 antibody was produced as previously described [45]. Antibodies to SUZ12 (Abcam), H3K4me2 (Upstate), EZH2 (Upstate), BMI1 (Upstate), RNA pol II (Covance), and MBD2 (Millipore) were purchased from commercial sources. The primer sequences and amplification conditions are available on request.

### ChIP-chip assay.

ChIP was combined with DNA microarray analysis to analyze HCT116 colorectal cancer cells using an antibody specific for trimethylation of histone H3 lysine 27 (H3K27me3) [82]*.* Using 5 × 10^7^ cells per experiment, ChIP, DNA amplification, labeling, and array hybridization were performed as previously described [83]. Whole-genome expanded promoter arrays from Agilent Technologies that span roughly 5 kb upstream to 2 kb downstream from gene transcription start sites were used. Enrichment values for each probe were calculated utilizing the default normalization in the Agilent Feature Extractor software.

### Transient siRNA transfection.

Tera-2 cells were transfected with either an NTC or EZH2 targeting siRNA (Dharmacon D-001210–01 and D-004218–01) using LipofectAMINE 2000 (Invitrogen). Cells were transfected at a 25 nmol/l siRNA concentration once and then transfected again 24 h later and continued to be passaged for up to 72 h.

### Methylation-specific PCRs (MSP).

We used the CpG island searcher program (http://cpgislands.usc.edu/) to identify CpG islands across the entire *GATA*-4 locus. The DNA extraction was performed using the Wizard Genomic DNA purification kit (Promega). MSP has been previously described [84]. One microgram of genomic DNA was used for bisulfite modification using the EZ methylation kit (Zymo Research) according to the manufacturer's protocol. MSP primers were designed using the MSPrimer [85] and were synthesized by Integrated DNA Technologies (Integrated DNA Technologies). The primer sequences and amplification conditions used for MSP analysis are available upon request.

## Supporting Information

Figure S1 Associated Chromosome Trap (ACT) Assay Discovers Remote Sequences in Close Physical Proximity with the *GATA-*4 Promoter in Undifferentiated Tera-2 Cells (A) The steps in the ACT assay and the results of the PCR reaction are shown. An example is shown where one intense band appears on a 2% agarose gel run on the product of the nested PCR reaction (top right). –L = no ligase, +L = ligase added, –A= no adapter, +A = adapter added.
(B) One example showing the sequence of the region (the linker sequence is not shown) that was trapped between the bait *Eco*RI proximal promoter site (underlined 3′ GAATTC sequence) and adapter ligated Msp I site (underlined 5′ CCGG sequence); the grey color sequence is the *GATA-*4 bait while the rest of the sequence is the captured fragment that comes from a region around 18.3 kb upstream of the proximal promoter *Eco*RI site.
(246 KB PDF)

Figure S2 The *ERCC*3 Locus Acquires a Stable Conformation in Undifferentiated Tera-2, Differentiated Tera-2, HCT116, and DKO Cells (A) Schematic representation of the location of *EcoR*I sites at the *ERCC*3 locus used for the 3C analysis. The distances of *EcoR*I sites are given with respect to the bait and not with respect to the transcription start site.
(B) The spatial organization of the *ERCC*3 locus in the four cell types examined HCT116, DKO, undifferentiated Tera-2, and differentiated Tera-2 cells. The graph is plotted as for Figure 1D (for explanation of symbols, refer to Figure 1D). Relative crosslinking frequencies of different regions with the bait fragment in undifferentiated Tera-2 cells (UT), differentiated Tera-2 (DT), HCT116, and DKO cells are shown as blue, purple, yellow, and green dots, respectively, as indicated to the far right of the panel.
(C) Realtime RT-PCR analysis for relative comparison of *ErCC3* transcript levels in the four cell types. The relative mRNA levels plotted on the *y*-axis were normalized to GAPDH.
(300 KB PDF)

Figure S3 Various Control Experiments for the 3C Assay (A) The *EcoR*I sites are digested to similar efficiency in undifferentiated Tera-2, differentiated Tera-2, HCT116, and DKO cells. We performed a PCR-based assay to determine digestion efficiency in the four cell types under investigation with the primers spanning some of the *EcoR*I sites used in the 3C analysis (−18.3, 0, −26.8, −4.6, −2.1, +35.6, +50.4, and +61.7) using the unligated DNA generated during the 3C procedure (where complete 3C assay was followed except that no ligase was added) (D). Similar amounts of undigested, unligated DNA from corresponding cell lines was run in parallel as a PCR control (U). UT= undifferentiated Tera-2 cells, DT= differentiated Tera-2 cells.
(B) Linear range of the 3C PCR amplification was determined for each sample prior to actual 3C amplifications. We carried out PCRs using different dilutions of 3C DNA using certain primer combinations (e.g., in this figure, PCRs done with one primer from −18.3 fragment and one primer from the promoter (0) fragment are shown). Similar titrations were done for each 3C sample to determine the initial quantity of the template to be used for 3C PCRs in order to make sure that the product formation takes place in the exponential phase of amplification.
(C) Control experiments were carried out using primer combinations that detect −18.3 and the promoter fragment interaction on several templates prepared with (+L) or without (–L) addition of ligase with various formaldehyde (HCHO) concentrations (from 0% to 2%). A BAC spanning the entire *GATA*-4 locus was digested and ligated to serve as a positive control for PCR. The PCR products are detected with the addition of both ligase and formaldehyde, and that too only to the intact nuclei and not to the genomic DNA, indicating that the ultimate products are a result of intramolecular ligation events resulting from specific DNA-protein interactions. In addition, the product formation increases linearly with the formaldehyde concentration.
(2.51 MB PDF)

Figure S4 DNA Segments Bound by H3K27me3 Were Defined Using ChIP and Followed by Hybridization of the DNA to Agilent Genomic Microarrays Containing Oligonucleotide Probes Spanning Promoter Regions of the Human Genome The bar graph shows normalized H3K27me3 ChIP enrichment ratios (calculated as the ratio of the signal for the bound fraction over the signal for the input or total DNA fraction) in HCT116 cells for all probes surrounding the *GATA*-4 transcription start site and are plotted against chromosomal position. The numbers on the *x*-axis represent the location of the probe (in base pairs) with respect to the transcription start site (0).
(150 KB PDF)

Figure S5 The Various Fragments across *GATA*-4 Locus Are Marked by Bivalent Marks in Undifferentiated Tera-2 Cells (A) Average enrichments for H3K4me2 and IgG (as control) for various fragments across the *GATA*-4 locus in undifferentiated and differentiated Tera-2 cells, from separate assays, are presented as the ratio of precipitated DNA (bound) to the total input DNA (1:100 dilution) and are plotted on the *y*-axis. Standard errors of the mean are indicated by the brackets. Black and grey bars represent enrichments for H3K4me2 in undifferentiated and differentiated Tera-2 cells respectively and white bars represent IgG control.
(B) The line graph compares the enrichments obtained above with H3K4me2 (in blue) and IgG (in yellow) to H3K27me3 enrichments (in pink) (from Figure 3B) in undifferentiated Tera-2 cells.
(165 KB PDF)

Figure S6 EZH2 Expression Is Higher in Adult Cancer Cell Lines as Compared to EC Cells Real-time RT-PCR analysis for relative comparison of EZH2 mRNA levels reveals that EZH2 transcripts are more abundant in colon cancer cells (HCT116 and DKO) than in any of the Tera-2 cells. It is also to be noted that the EZH2 is several fold down-regulated on differentiation of Tera-2 cells as shown previously [38], which accompanies resolution of the chromatin higher order structure at the *GATA*-4 locus (Figure 1C and 1D). Total RNA was prepared from HCT116, DKO, undifferentiated Tera-2 cells, and differentiated Tera-2 cells using the RNeasy Kit (Qiagen), followed by cDNA synthesis and real-time PCR using QuantiTect SYBER Green PCR Kit (Qiagen). UT= undifferentiated Tera-2 cells, DT= differentiated Tera-2 cells. The numbers on top of the bars indicate fold upregulation in EZH2 mRNA levels for DT, HCT116, and DKO cells as compared to the value for the UT cells, which is set to 1.
(116 KB PDF)

Figure S7 The *GATA-*4 Locus is Moderately Enriched with Polycomb Proteins and the Associated Mark H3K27me3 in HCT116 and DKO Cells (A) Following ChIP assay, the quantitation was done exactly as described for Figure 3B and 3C. Average enrichments for EZH2 (upper panel), H3K27me3 (lower panel), and IgG (as control) from separate assays are presented as the ratio of precipitated DNA (bound) to the total input DNA (1:100 dilution) for various fragments across the entire *GATA*-4 locus and are plotted on the *y*-axis. Standard errors of the mean are indicated by the brackets. Black bars represent HCT116 cells, grey bars represent DKO cells, and white bars represent IgG control.
(B) Similar analysis was performed for EZH2 (upper panel) and H3K27me3 (lower panel) enrichments in HCT116 and DKO cells for various fragments spread across the *CDKN2B* (p15) locus in HCT116 and DKO cells. The graphs are plotted exactly as in (A).
(442 KB PDF)

Figure S8 Click here for additional data file. MBD2 Is Enriched at all Six Methylated CpG Islands across the GATA-4 Locus Only in HCT116 Cells and Not in the DKO Cells
ChIP was performed using antibody against MBD2, and the precipitated DNA was amplified using primers specific for various CpG islands in HCT116 and DKO cells. Enrichments are presented as folds of total input and are compared with the control samples (no antibody and IgG).
(131 KB PDF)

Figure S9 A Model of How *GATA*-4 Locus Might Be Organized in Undifferentiated Tera-2 Cells By proposing this model, we also raise the possibility that the one of the chromatin regions distal to the proximal promoter acts as a recruitment centre for PcG proteins. Examples of such fragments are those located at −31.3 and −18.3 kb, which interact with a high frequency with many fragments throughout the locus including the promoter (Figure 1B and 1D) and also show very strong enrichments for PcG proteins such as EZH2 and SUZ12 as well as the associated repressive mark H3K27me3 (Figure 3B–3D). This initial recruitment at the distal 5′ region is followed by progressive interaction of the PcG-bound distal element with the target promoter most probably via looping or linking mechanisms [57] to establish transcriptional silencing. The loading of PcG proteins and the deposition of H3K27me3 marks in and around other regions and establishment of long-range interactions are a consequence of such action. Similar dynamics could also occur for fragments at the 3′ region (not depicted here) and the observations made using the 3C assay, shown in Figure 6, represent the steady-state situation of this process.
(282 KB PDF)

## Abbreviations

3C: chromosome conformation capture
ACT: associated chromosome trap
ChIP: chromatin immunoprecipitation
EC: embryonic carcinoma
ES: embryonic stem
MSP: methylation-specific PCR
NTC: non-targeting control
PcG: Polycomb group
PRE: Polycomb Response Element
pre-RCH: pre-repressive chromatin hub
