# Supplementary material for: PcG Proteins, DNA Methylation, and Gene Repression by Chromatin Looping
Source: PLoS Biol. 2008 Dec 2;6(12):e306. doi: 10.1371/journal.pbio.0060306 (PMC2592355; doi:10.1371/journal.pbio.0060306)
Supplement: Supplementary file 1 [file pbio.0060306.sg001.pdf]

A

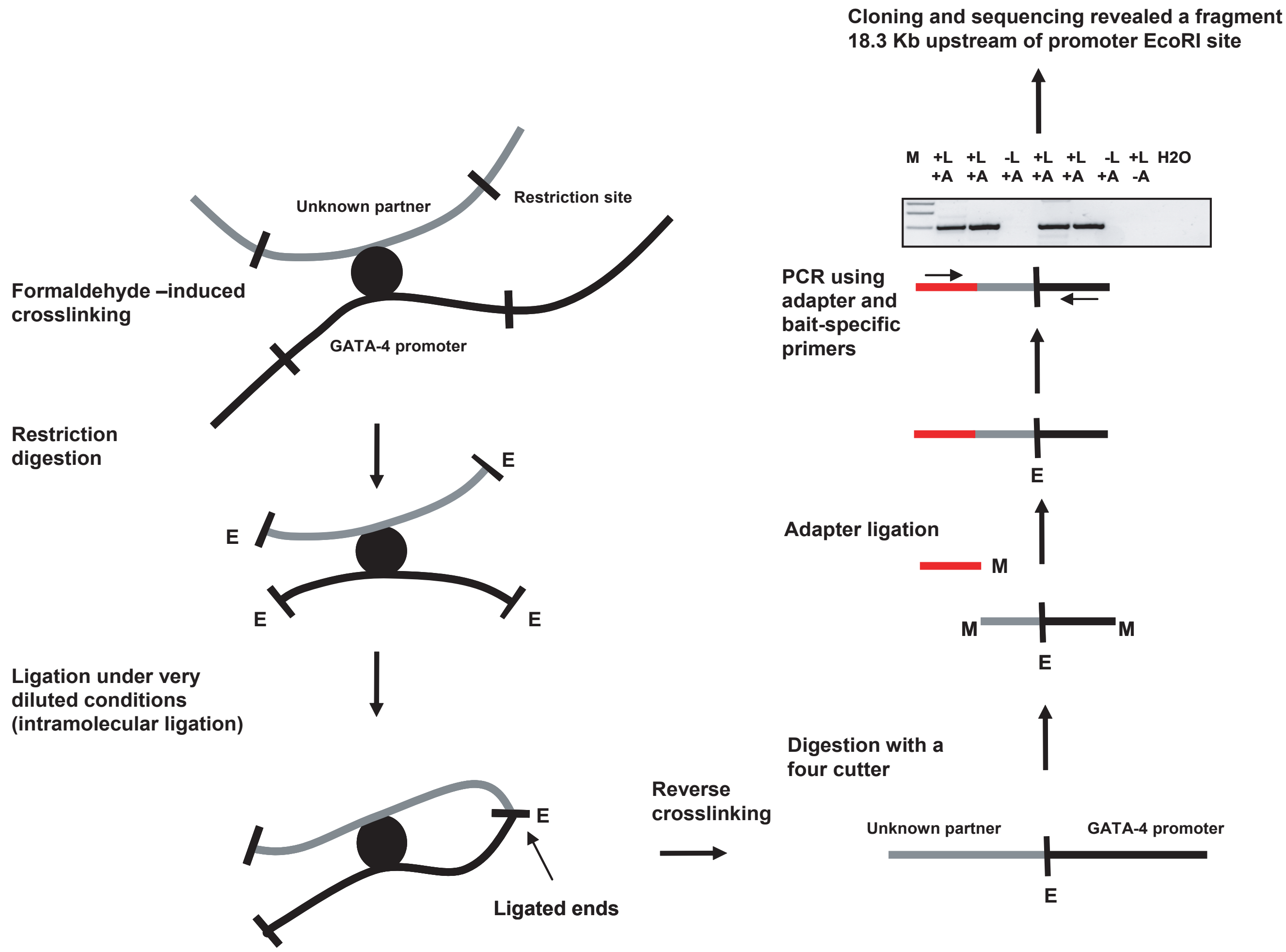

B

ccgggtttatgccccaaaagtaagagttcccctgcccagggcaacccatgtcctcagccataactggctggccacgacctgggctgtgccatggctcccttcagccttgccctggtgatggagatcc
ctgagctgctagaaactaaagcccctgaaacctgaagacgctgtgggccttagtcaagccaaccttcctctgatgttatggtggatatttgctgccaggtgagagaaaacactacagggagtagcca
aagaattgccagagcctagattctagattctagttcccacacaaacacctacaagctgggtgaccttgagtaagttatttcacttctatttctagcatagtctaaaaataaggtgcaggaatgtgcttctcaa
aagttgaaaatgacatctttttcaaagataaaatttctccttatagtctgtgttcacaaaagccaatttctgaaccataaactcattacaaagaatgttctggaaaccttttagatttgtttagatcctaaatgtac
atcgtccctaggtcagtgtaagagtttagactccagtattagaatgttgtgatttgaattccccgcgcagccgcgctcgtttcttcctctgggattttcctgagaatccccaggagttggc
