# Supplementary material for: PcG Proteins, DNA Methylation, and Gene Repression by Chromatin Looping
Source: PLoS Biol. 2008 Dec 2;6(12):e306. doi: 10.1371/journal.pbio.0060306 (PMC2592355; doi:10.1371/journal.pbio.0060306)
Supplement: Supplementary file 2 [file pbio.0060306.sg002.pdf]

A

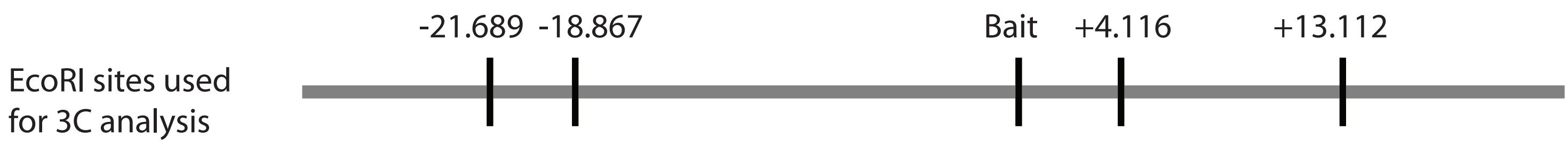

-21.689
-18.867
Bait
+4.116
+13.112
EcoRI sites used for 3C analysis

B

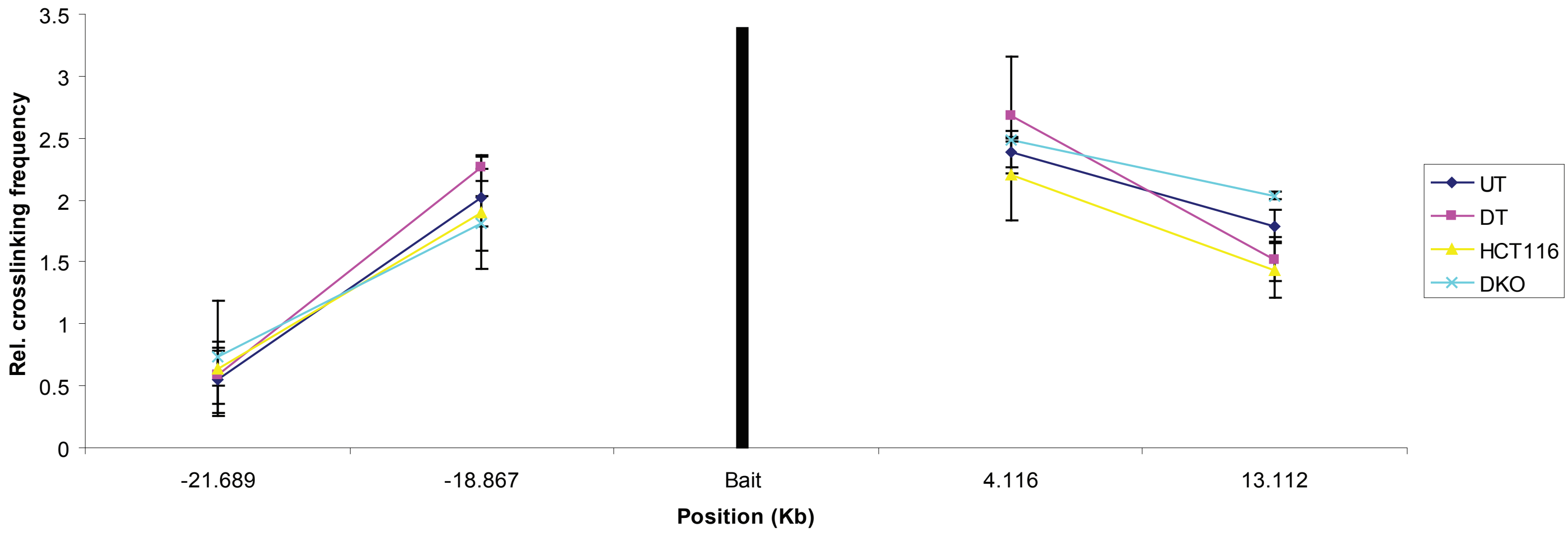

Rel. crosslinking frequency
3.5
3
2.5
2
1.5
1
0.5
0
-21.689
-18.867
Bait
4.116
13.112
Position (Kb)
UT
DT
HCT116
DKO

C

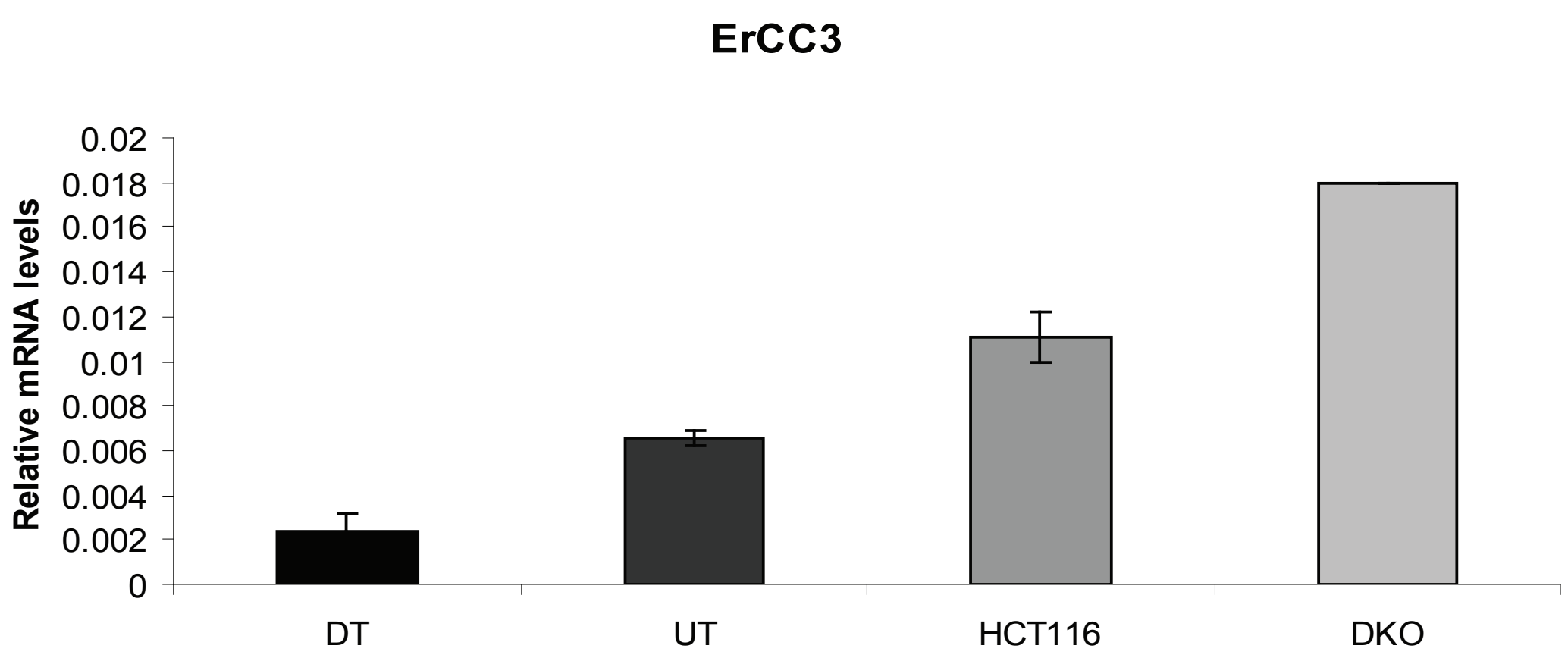

ErCC3
Relative mRNA levels
0.02
0.018
0.016
0.014
0.012
0.01
0.008
0.006
0.004
0.002
0
DT
UT
HCT116
DKO
