# Supplementary material for: PcG Proteins, DNA Methylation, and Gene Repression by Chromatin Looping
Source: PLoS Biol. 2008 Dec 2;6(12):e306. doi: 10.1371/journal.pbio.0060306 (PMC2592355; doi:10.1371/journal.pbio.0060306)
Supplement: Supplementary file 3 [file pbio.0060306.sg003.pdf]

A.

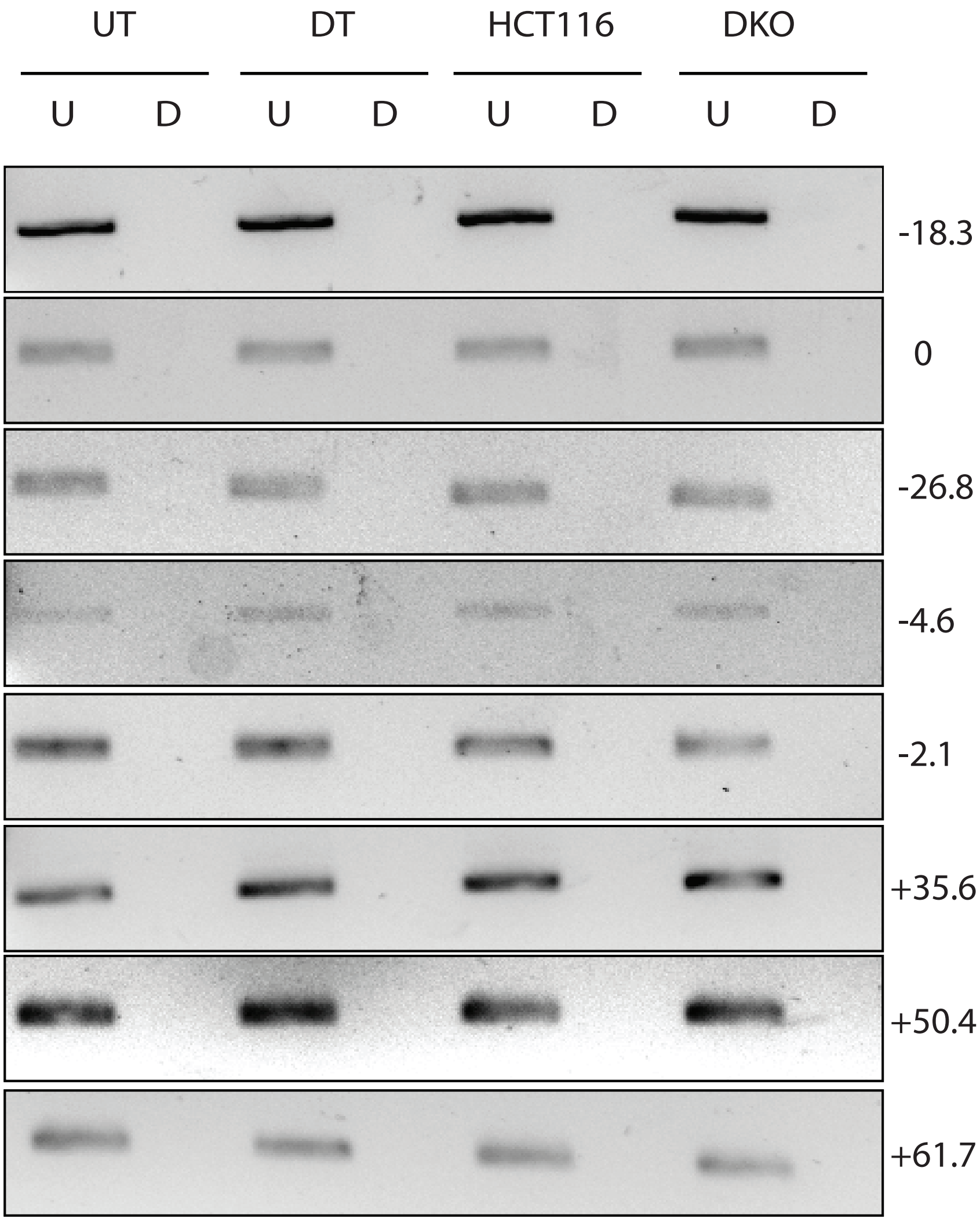
UT
DT
HCT116
DKO
U D U D U D U D
-18.3
0
-26.8
-4.6
-2.1
+35.6
+50.4
+61.7

B.

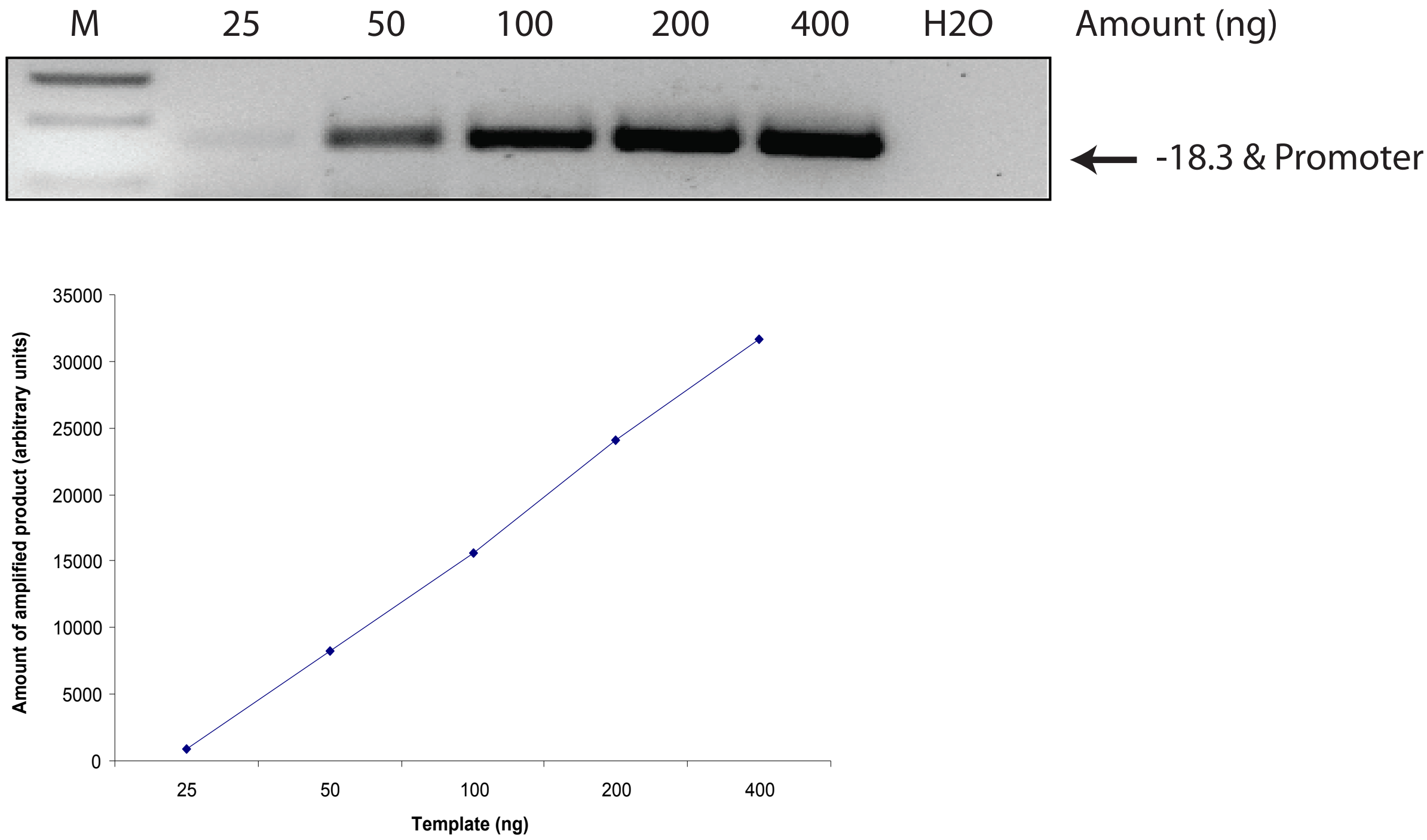
M
25
50
100
200
400
H2O
Amount (ng)
-18.3 & Promoter
Amount of amplified product (arbitrary units)
35000
30000
25000
20000
15000
10000
5000
0
25
50
100
200
400
Template (ng)

C.

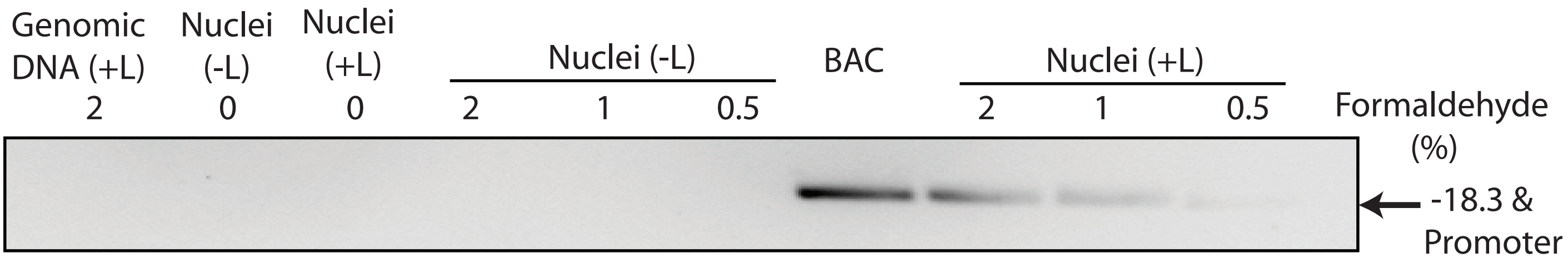
Genomic DNA (+L)
Nuclei (-L)
Nuclei (+L)
Nuclei (-L)
BAC
Nuclei (+L)
2
0
0
2
1
0.5
2
1
0.5
Formaldehyde (%)
-18.3 & Promoter
