# Supplementary material for: PcG Proteins, DNA Methylation, and Gene Repression by Chromatin Looping
Source: PLoS Biol. 2008 Dec 2;6(12):e306. doi: 10.1371/journal.pbio.0060306 (PMC2592355; doi:10.1371/journal.pbio.0060306)
Supplement: Supplementary file 4 [file pbio.0060306.sg004.pdf]

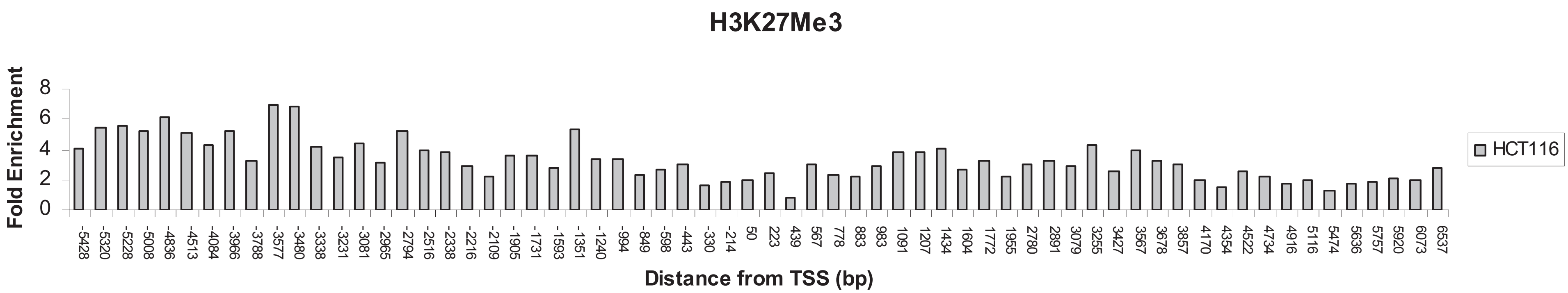
H3K27Me3
Fold Enrichment
8
6
4
2
0
-5428
-5320
-5228
-5008
-4836
-4513
-4084
-3966
-3788
-3577
-3480
-3338
-3231
-3081
-2965
-2794
-2516
-2338
-2216
-2109
-1905
-1731
-1593
-1351
-1240
-994
-849
-598
-443
-330
-214
50
223
439
567
778
883
983
1091
1207
1434
1604
1772
1955
2780
2891
3079
3255
3427
3567
3678
3857
4170
4354
4522
4734
4916
5116
5474
5636
5757
5920
6073
6537
Distance from TSS (bp)
HCT116
