# Supplementary material for: PcG Proteins, DNA Methylation, and Gene Repression by Chromatin Looping
Source: PLoS Biol. 2008 Dec 2;6(12):e306. doi: 10.1371/journal.pbio.0060306 (PMC2592355; doi:10.1371/journal.pbio.0060306)
Supplement: Supplementary file 5 [file pbio.0060306.sg005.pdf]

A.

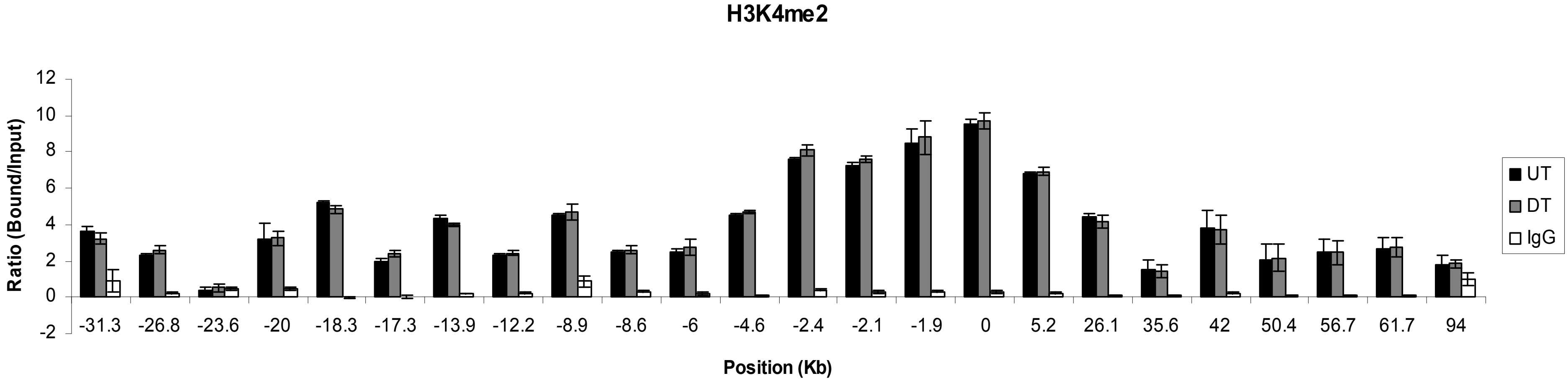
H3K4me2
Ratio (Bound/Input)
12
10
8
6
4
2
0
-2
-31.3
-26.8
-23.6
-20
-18.3
-17.3
-13.9
-12.2
-8.9
-8.6
-6
-4.6
-2.4
-2.1
-1.9
0
5.2
26.1
35.6
42
50.4
56.7
61.7
94
Position (Kb)
UT
DT
IgG

B.

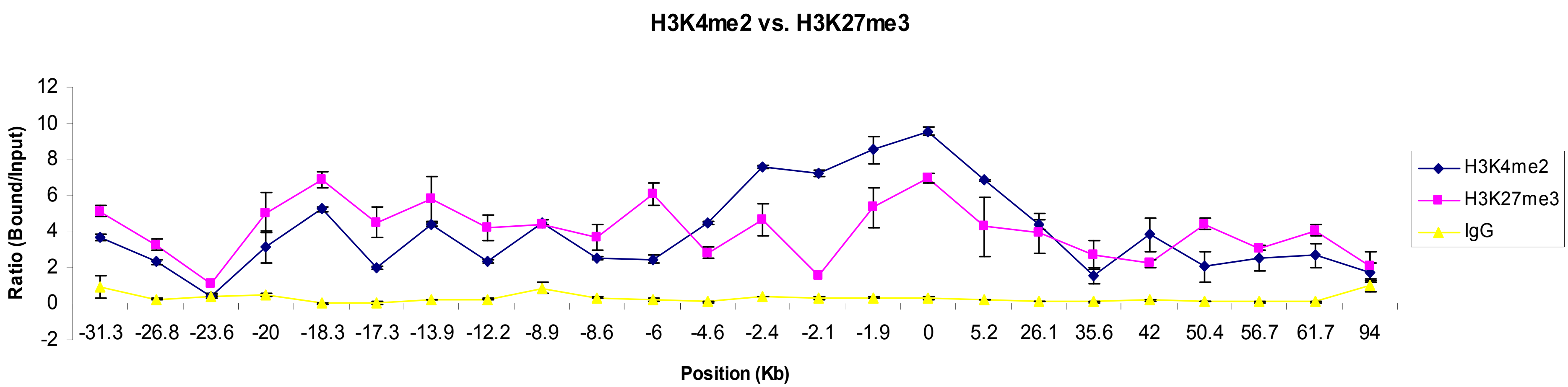
H3K4me2 vs. H3K27me3
Ratio (Bound/Input)
12
10
8
6
4
2
0
-2
-31.3
-26.8
-23.6
-20
-18.3
-17.3
-13.9
-12.2
-8.9
-8.6
-6
-4.6
-2.4
-2.1
-1.9
0
5.2
26.1
35.6
42
50.4
56.7
61.7
94
Position (Kb)
H3K4me2
H3K27me3
IgG
