# Supplementary material for: PcG Proteins, DNA Methylation, and Gene Repression by Chromatin Looping
Source: PLoS Biol. 2008 Dec 2;6(12):e306. doi: 10.1371/journal.pbio.0060306 (PMC2592355; doi:10.1371/journal.pbio.0060306)
Supplement: Supplementary file 6 [file pbio.0060306.sg006.pdf]

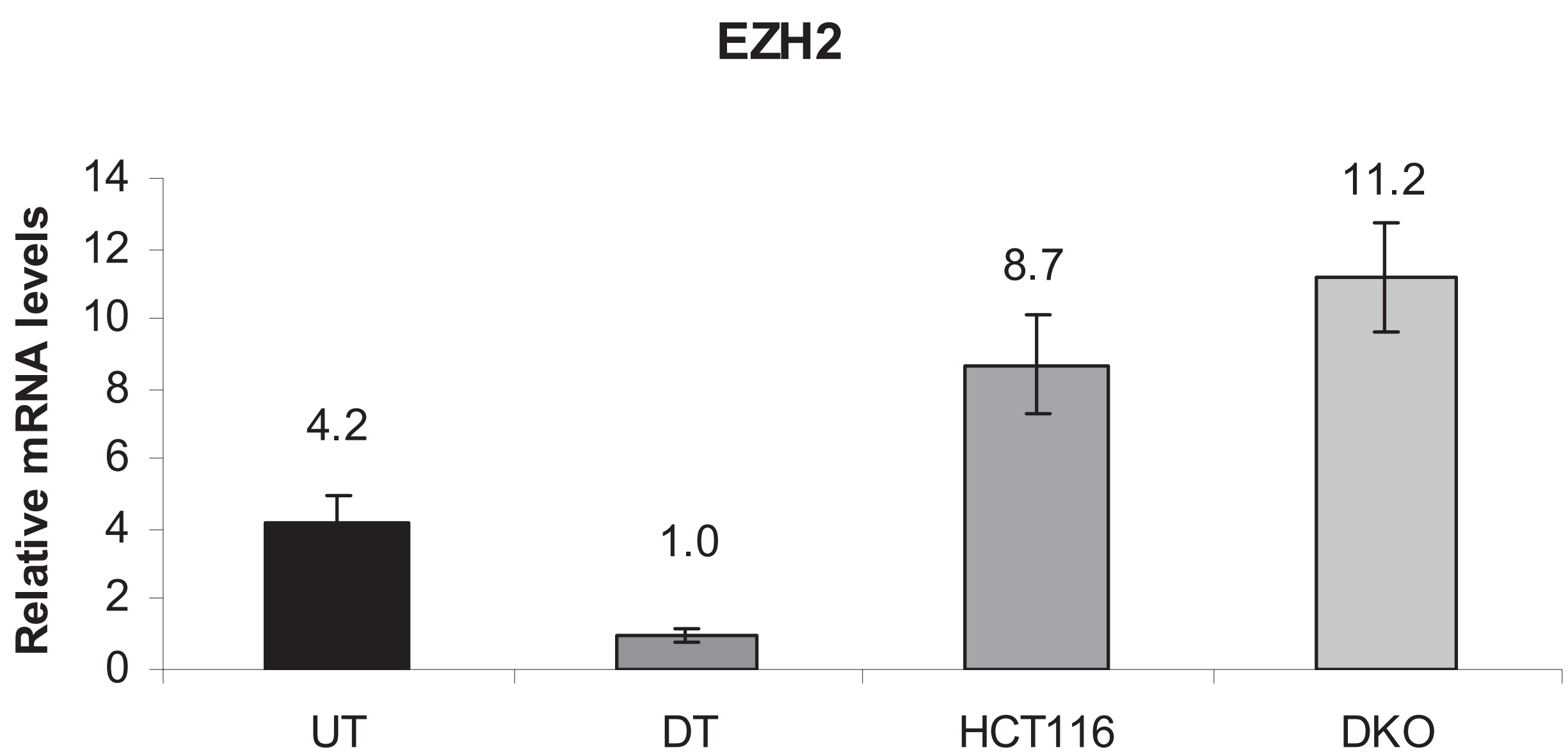
EZH2
Relative mRNA levels
14
12
10
8
6
4
2
0
4.2
1.0
8.7
11.2
UT
DT
HCT116
DKO
