# Supplementary material for: PcG Proteins, DNA Methylation, and Gene Repression by Chromatin Looping
Source: PLoS Biol. 2008 Dec 2;6(12):e306. doi: 10.1371/journal.pbio.0060306 (PMC2592355; doi:10.1371/journal.pbio.0060306)
Supplement: Supplementary file 7 [file pbio.0060306.sg007.pdf]

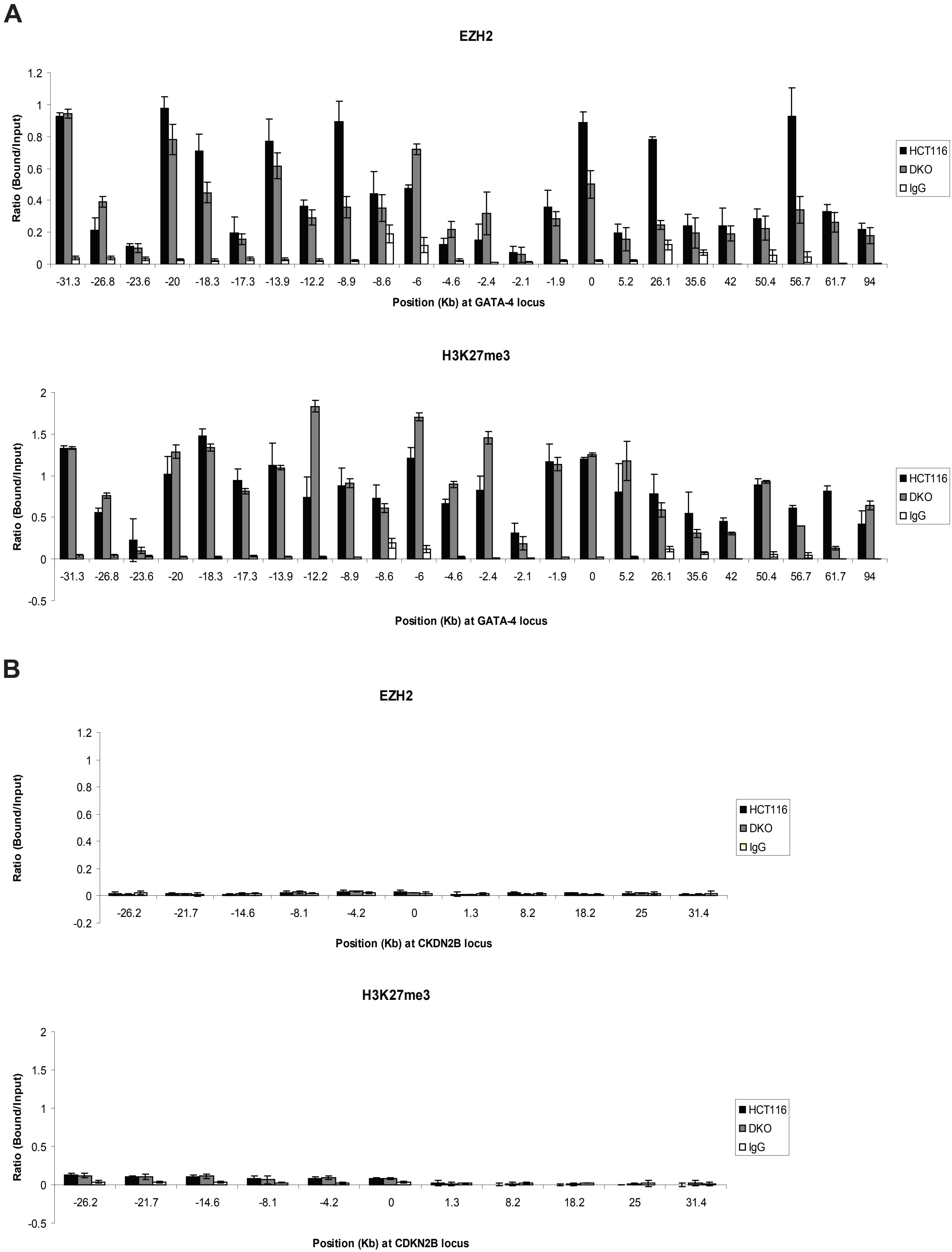
A
EZH2
Ratio (Bound/Input)
1.2
1
0.8
0.6
0.4
0.2
0
-31.3 -26.8 -23.6 -20 -18.3 -17.3 -13.9 -12.2 -8.9 -8.6 -6 -4.6 -2.4 -2.1 -1.9 0 5.2 26.1 35.6 42 50.4 56.7 61.7 94
Position (Kb) at GATA-4 locus
HCT116
DKO
IgG
H3K27me3
Ratio (Bound/Input)
2
1.5
1
0.5
0
-0.5
-31.3 -26.8 -23.6 -20 -18.3 -17.3 -13.9 -12.2 -8.9 -8.6 -6 -4.6 -2.4 -2.1 -1.9 0 5.2 26.1 35.6 42 50.4 56.7 61.7 94
Position (Kb) at GATA-4 locus
HCT116
DKO
IgG
B
EZH2
Ratio (Bound/Input)
1.2
1
0.8
0.6
0.4
0.2
0
-0.2
-26.2 -21.7 -14.6 -8.1 -4.2 0 1.3 8.2 18.2 25 31.4
Position (Kb) at CKDN2B locus
HCT116
DKO
IgG
H3K27me3
Ratio (Bound/Input)
2
1.5
1
0.5
0
-0.5
-26.2 -21.7 -14.6 -8.1 -4.2 0 1.3 8.2 18.2 25 31.4
Position (Kb) at CDKN2B locus
HCT116
DKO
IgG
