# Supplementary material for: PcG Proteins, DNA Methylation, and Gene Repression by Chromatin Looping
Source: PLoS Biol. 2008 Dec 2;6(12):e306. doi: 10.1371/journal.pbio.0060306 (PMC2592355; doi:10.1371/journal.pbio.0060306)
Supplement: Supplementary file 8 [file pbio.0060306.sg008.pdf]

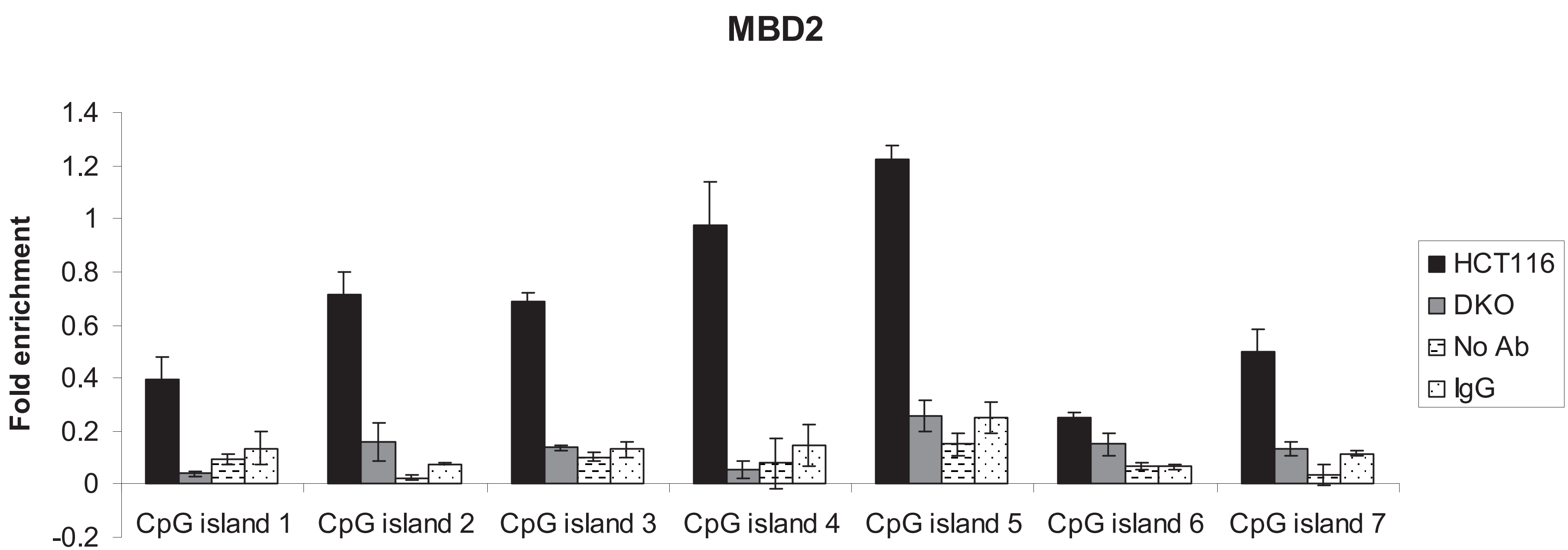

MBD2
Fold enrichment
1.4
1.2
1
0.8
0.6
0.4
0.2
0
-0.2
CpG island 1
CpG island 2
CpG island 3
CpG island 4
CpG island 5
CpG island 6
CpG island 7
HCT116
DKO
No Ab
IgG
