# Supplementary material for: PcG Proteins, DNA Methylation, and Gene Repression by Chromatin Looping
Source: PLoS Biol. 2008 Dec 2;6(12):e306. doi: 10.1371/journal.pbio.0060306 (PMC2592355; doi:10.1371/journal.pbio.0060306)
Supplement: Supplementary file 9 [file pbio.0060306.sg009.pdf]

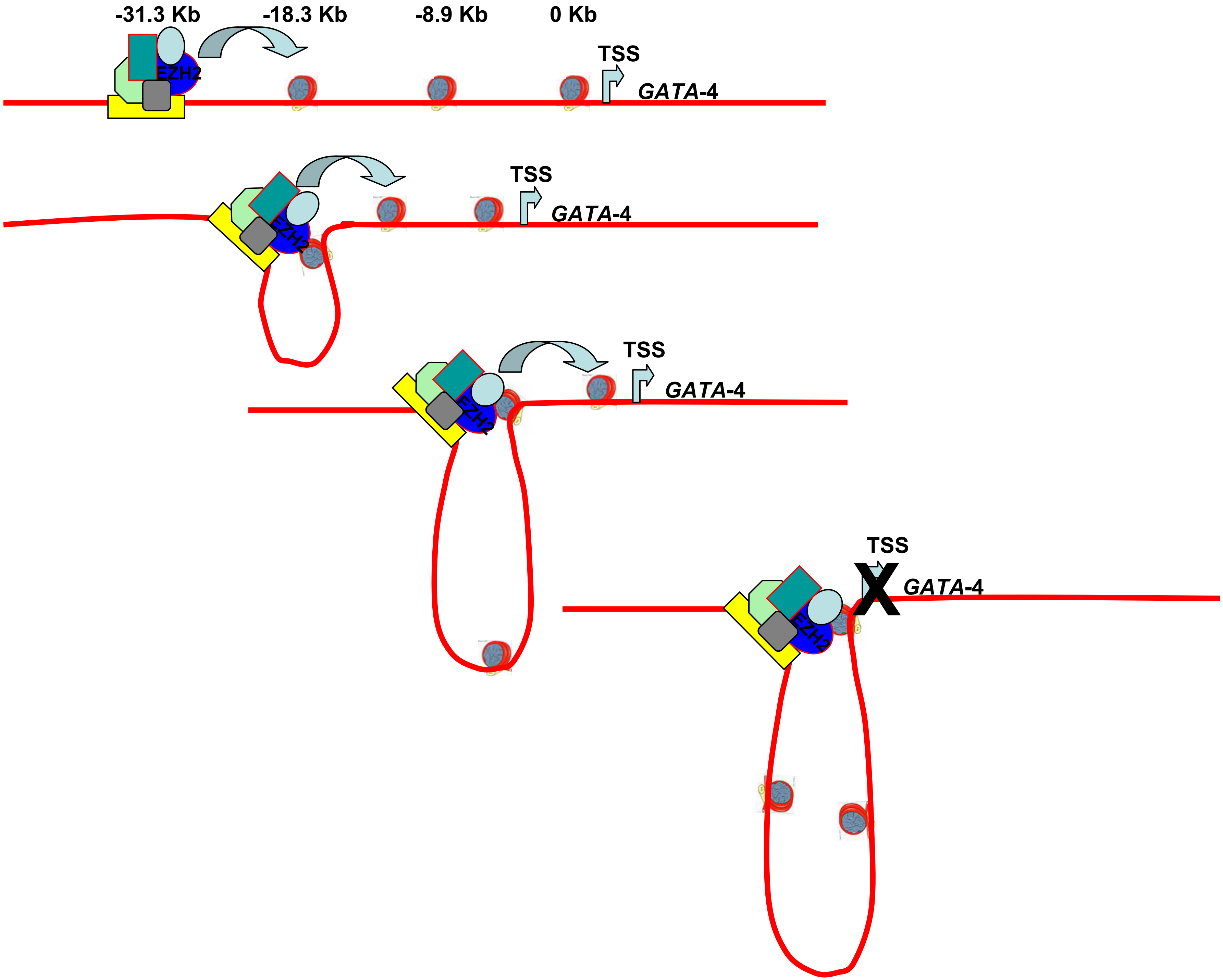

-31.3 Kb
-18.3 Kb
-8.9 Kb
0 Kb
TSS
GATA-4
EZH2
TSS
GATA-4
EZH2
TSS
GATA-4
EZH2
TSS
GATA-4
EZH2
